# Biofluid biomarker changes following treatment with sabirnetug (ACU193) in INTERCEPT-AD, a phase 1 trial in early Alzheimer's disease

**DOI:** 10.1016/j.tjpad.2025.100082

**Published:** 2025-02-17

**Authors:** Erika N. Cline, Daniel Antwi-Berko, Karen Sundell, Elizabeth Johnson, Maddelyn Hyland, Hao Zhang, Hugo Vanderstichele, June Kaplow, Robert A. Dean, Erik Stoops, Eugeen Vanmechelen, Marleen J.A. Koel-Simmelink, Charlotte E. Teunissen, Gopalan Sethuraman, Todd Feaster, Eric Siemers, Jasna Jerecic

**Affiliations:** a Acumen Pharmaceuticals, Inc, 1210-1220 Washington St., Suite 210, Newton, MA 02465, USA; b Neurochemistry Laboratory, Department of Laboratory Medicine, Amsterdam UMC, Amsterdam, the Netherlands; c ADx NeuroSciences, Technologiepark 6, Gent, Belgium

**Keywords:** ACU193, Sabirnetug, Alzheimer's disease, Biomarkers, Cerebrospinal fluid

## Abstract

**Objective:**

Sabirnetug (ACU193) is a humanized monoclonal antibody selective for soluble amyloid beta oligomers (AβOs), synaptotoxins that are early and persistent triggers of Alzheimer's disease (AD). Sabirnetug pharmacodynamics were examined in the INTERCEPT-AD phase 1 study in mild cognitive impairment and mild dementia due to AD (NCT04931459) using biofluid biomarkers associated with Aβ and tau pathology, synaptic dysfunction, neuroinflammation, and neurodegeneration.

**Methods:**

INTERCEPT-AD was a randomized, first-in-human study of sabirnetug versus placebo administered as a single (SAD; 2, 10, 25, 60 mg/kg) or multiple (MAD; three doses of 10 or 60 mg/kg every 4 weeks [Q4W] or 25 mg/kg Q2W) ascending doses. Biomarkers were measured pre-/post-dose in CSF and EDTA-plasma. Correlations of biomarker changes versus dose, exposure duration, and target engagement were determined.

**Results:**

In MAD cohorts, CSF pTau181 decreased significantly (60 mg/kg Q4W, *p* = 0.049). VAMP2 decreased significantly at all doses (*p* ≤ 0.041); neurogranin decreased significantly at 60 mg/kg Q4W (*p* = 0.037). Aβ_1-42_/Aβ_1–40_ trended upward with sabirnetug dose. Aβ_1–42_/Aβ_1–40_ and neurogranin changes correlated with sabirnetug-AβO target engagement (*p* ≤ 0.01). Decreases in tTau, VAMP2, and neurogranin correlated with exposure duration (*p* ≤ 0.007). Plasma pTau181, pTau217, GFAP, and NfL trended lower.

**Discussion:**

Following three sabirnetug doses, changes in CSF and plasma biomarkers were observed. The CSF biomarker response increased with increasing dose and exposure duration, consistent with previous reports that sabirnetug reaches the central compartment and engages its AβO target. The ongoing phase 2 ALTITUDE-AD study (NCT06335173) will test whether sabirnetug's pharmacodynamic effects can be substantiated with a larger sample size and longer treatment duration.

## 1 Introduction

Soluble species of amyloid beta (Aβ) peptides, including globular Aβ oligomers (AβOs) and linear Aβ protofibrils, have been shown to impair synaptic function and are posited to be the most pathogenic form of Aβ and a significant causal factor in Alzheimer's disease (AD) [[1], [2], [3], [4], [5], [6], [7], [8]]. Monoclonal antibodies (mAbs) that selectively target and/or block the formation of AβOs have shown promise for AD drug development and treatment [9]. Sabirnetug (ACU193) is a humanized, affinity-matured, immunoglobulin G2 subclass monoclonal antibody raised against globular species of AβOs [1,10]. Sabirnetug has at least a 650-fold greater binding affinity for AβOs in a competitive ELISA assay than for Aβ monomers and appears to show limited binding to amyloid plaques [1].

The safety, tolerability, pharmacokinetics (PK), and pharmacodynamic (PD) activity of sabirnetug in patients with mild cognitive impairment (MCI) and mild dementia due to AD was recently tested in INTERCEPT-AD, a phase 1, placebo-controlled, single- and multiple-ascending dose clinical trial (NCT04931459) [11,12]. Primary results, including demographics, clinical safety, tolerability, PK, PD (assessed by amyloid plaque load using florbetapir positron emission tomography [PET] imaging) and target engagement as determined in CSF (sabirnetug bound to AβOs) have been previously published [11]. Sabirnetug was well-tolerated with intravenous (IV) dosing up to 12 weeks. Sabirnetug exposure observed with multiple ascending doses (MAD) was generally dose-proportional and similar to that observed in single ascending dose (SAD) cohorts. Three infusions of the two highest doses (60 mg/kg every four weeks [Q4W] or 25 mg/kg every 2 weeks [Q2W]) resulted in significant reduction from baseline in amyloid PET Centiloid values (25 % and 20 %, respectively) [11].

**Fig. 1:**
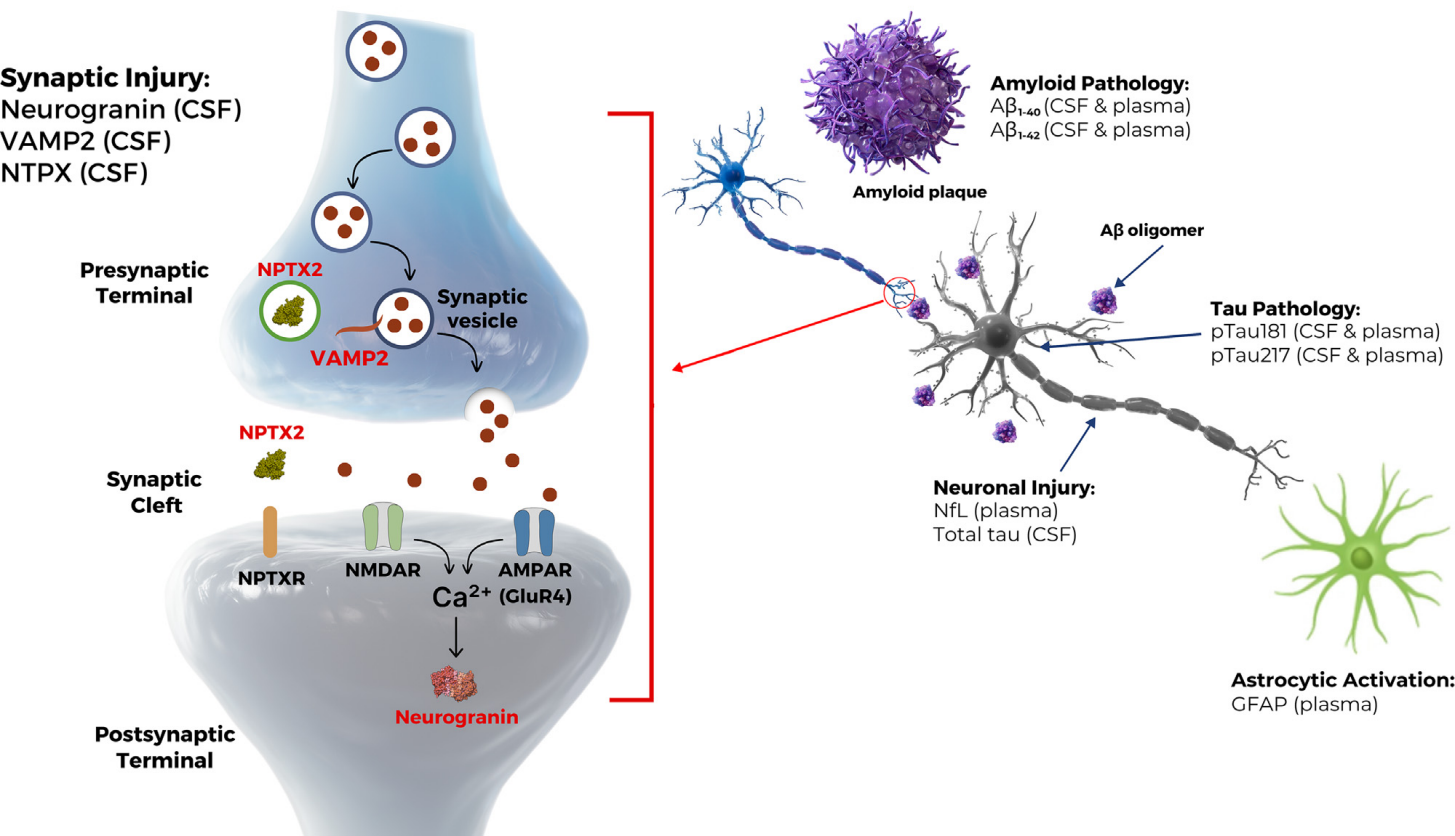
Key CSF Biomarkers Associated with Pathology of Alzheimer's Disease. The synaptic portion of the figure is adapted from Das et al., Alzheimer's Res Ther, 2023;15:62. https://doi.org/10.1186/s13195-023-01212-x per CC-BY copyright to highlight biomarkers measured in INTERCEPT-AD. https://creativecommons.org/licenses/by/4.0/.

In INTERCEPT-AD, multiple ATX(N) biomarkers (*A* = Aβ pathway, *T* = tau-mediated pathophysiology, *X* = additional pathophysiological mechanisms such as synaptic dysfunction, N = neurodegeneration) [13,14] were measured in CSF and EDTA-plasma as exploratory measures of PD activity [11]. The selected biomarkers are shown in Fig. 1. Changes in the Aβ, tau, and neurodegenerative biomarkers are associated with the progression of AD pathology [15]. Due to sabirnetug's selectivity for AβOs and its potential to interrupt AβO binding to synapses, changes in synaptic biomarkers were also assessed, specifically the pre-/post-synaptic protein vesicle associated membrane protein 2 (VAMP2), pre-synaptic neuronal pentraxin 2 (NPTX2), and post-synaptic neurogranin. Increases in VAMP2 and neurogranin in CSF have been shown to be associated with synaptic degeneration and AD progression [[16], [17], [18]]. Decreases in CSF NPTX2 concentrations have been observed in patients with AD or MCI compared to cognitively normal controls [[19], [20], [21], [22], [23]]. However, CSF NPTX2 concentrations have also been shown to increase in individuals who are within 2 years of MCI symptom onset [[16], [17], [18]]. Thus, its potential as a biomarker of synaptic function in AD remains to be fully elucidated. Other biomarkers of potential interest in AD include neurofilament light (NfL) and glial fibrillary acidic protein (GFAP). NfL is a structural protein primarily within myelinated axons/neurons; high concentrations in CSF and blood are associated with neurodegeneration and neuronal injury [[19], [20], [21], [22], [23]]. GFAP is associated with astrocytic activity and also appears to be increased during neurodegeneration [24].

This report presents analyses of the ATX(N) CSF and plasma biofluid biomarkers used as exploratory measures of PD activity following one to three doses of sabirnetug in the INTERCEPT-AD study.

## 2 Methods

INTERCEPT-AD (NCT04931459) was a phase 1, randomized, double-blind, first-in-human study of sabirnetug conducted at 15 sites in the United States in participants with early symptomatic AD (MCI or mild dementia due to AD). Trial design as well as safety (primary objective), PK (secondary objective), and preliminary PD findings (exploratory PD objective) and participant demographics have been previously reported [11]. Briefly, INTERCEPT-AD part A (SAD cohorts) included four cohorts of participants randomized to receive a single IV infusion of sabirnetug or placebo in a 6:2 ratio (sabirnetug [*n* = 6/cohort] and placebo [*n* = 2/cohort]). Sabirnetug-treated participants in the SAD cohorts received: 2 mg/kg, 10 mg/kg, 25 mg/kg, or 60 mg/kg sabirnetug. In Part B (MAD cohorts) of INTERCEPT-AD, three cohorts of participants were randomized to receive three IV infusions of sabirnetug or placebo in an 8:2 ratio (sabirnetug [*n* = 8/cohort] and placebo [*n* = 2/cohort]). Dosing regimens for participants in MAD cohorts were three doses of 10 mg/kg or 60 mg/kg sabirnetug or placebo every four weeks (Q4W) or 25 mg/kg sabirnetug or placebo every two weeks (Q2W). An exploratory PD objective of this study was to evaluate changes in CSF and blood-based concentrations of biomarkers associated with AD or other neurodegenerative disease following single- and multiple-dose administration of sabirnetug.

### 2.1 Ethics

INTERCEPT-AD was conducted in accordance with the protocol, International Council for Harmonisation (ICH) E6 Good Clinical Practice (GCP), the Declaration of Helsinki (2008), and all applicable regulatory requirements. The protocol and informed consent form were approved by ethics committees at all study sites and written informed consent was provided by all participants or their legally-authorized representatives prior to engaging in study activities.

### 2.2 Sample collection, performing laboratories and quality assurance

An abbreviated Study Schedule showing the nominal timing of dose administration versus CSF collection by lumbar puncture (LP) and EDTA-plasma collection is shown in Supplemental Table 1. The actual CSF and plasma collection time could vary from the nominal time due to participant visit scheduling variation. For CSF collection in MAD cohorts specifically, the actual time between the first dose and the endpoint CSF collection was 30–47 days in the Q2W group and 62–92 days in the Q4W groups. CSF pTau217 was analyzed by ADx NeuroSciences, Gent, Belgium. All other biomarker analyses were performed by the Neurochemistry Lab of Amsterdam UMC, The Netherlands. For all analyses, the performing labs qualified the assay method as fit-for-purpose prior to use in this study. CSF and plasma drug interference was evaluated commensurate to measured drug exposure duration (defined as the time between first dose and endpoint LP) of sabirnetug. Documentation of inter-laboratory standardization methods and quality assurance procedures are provided in the Supplemental Materials.

### 2.3 CSF assays

Characteristics of assays used for CSF biomarkers are contained in Supplemental Table 2. Aβ_1–42_ was measured using the Lumipulse G β-Amyloid 1–42 assay manufactured by Fujirebio Inc. (Tokyo, Japan) (catalog # FRI38390). Aβ_1–40_ was measured using the Lumipulse G β-Amyloid 1–40 immunoreaction cartridges manufactured by Fujirebio Inc. (Tokyo, Japan) (catalog # FRI00103). tTau was measured using the Lumipulse G tTau immunoreaction cartridges manufactured by Fujirebio Inc. (Tokyo, Japan) (catalog # FRI00075). pTau181 was measured using the Lumipulse G (1200) pTau181 immunoreaction cartridges manufactured by Fujirebio Inc. (Tokyo, Japan) (catalog # FRI00064). Neurogranin, C-terminally truncated at P75, was measured using the Neurogranin ELISA kit manufactured by EUROIMMUN AG (Lubeck, Germany) (catalog # EQ 6551–9601-L). NPTX2 was measured using the INNOTEST NPTX2 ELISA kit manufactured by Fujirebio Europe N.V. (Ghent, Belgium) (catalog # 80908). pTau217 was measured using a prototype assay on the Lumipulse G1200 platform. ADx NeuroSciences performed a limited analytical characterization of this prototype assay method prior to its use in this study. CSF samples were manually diluted 4-fold and measured in singlicate within the measurement range of the assay (0.02–25.6 pg/mL). All sample concentrations were within the quantifiable range of the assay at this 4-fold dilution except for 4 samples that needed further dilution of 20- and 40-fold. Therefore, parallelism was demonstrated up to 40-fold dilution (within the range of 80–120 % after back calculation of the obtained concentration) using remnant CSF samples. Furthermore, the assay demonstrated an inter-run precision between 2.5 % CV and 11.8 % CV (testing of 3 neat CSF remnant samples with low, medium and high concentrations in singlicate over 4 consecutive test runs).

VAMP2 was measured using a prototype ELISA assay developed by ADx NeuroSciences. The performing Neurochemistry laboratory at Amsterdam UMC qualified the assay method prior to its use in this study; all sample results were within the quantifiable range of the assay.

All CSF samples were tested within three freeze-thaw cycles.

### 2.4 EDTA-plasma assays

Assays used for plasma biomarkers are contained in Supplemental Table 3.

Aβ_1–42_, Aβ_1–40_, GFAP, and NfL were measured in EDTA-plasma using the Simoa® Neurology 4-plex E (N4PE) Advantage kit manufactured by the Quanterix Corporation (Billerica, MA) (catalog # 103,670). pTau181 was measured in participant EDTA-plasma using the Simoa® pTau-181 Advantage V2.1 kit manufactured by the Quanterix Corporation (Billerica, MA) (catalog # 104111 of 103,714). pTau217 was measured in participant EDTA-plasma using the Simoa® Alzpath pTau-217 CARe Advantage kit manufactured by the Quanterix Corporation (Billerica, MA) (MAB 231122). All of these assays were measured on the Simoa HD-X platform. All plasma samples were tested within two freeze-thaw cycles.

### 2.5 Statistical methods

All analyses and tabulations were performed using SAS Enterprise Guide, version 8.3 and graphs were produced using GraphPad Prism version 10. Summary statistics on baseline to endpoint changes in concentrations of CSF and plasma biomarkers by cohort were obtained comparing pre-dose and post-dose values. The intention-to-treat (ITT) population, defined as all randomized participants who received at least one infusion, was used in these analyses. The duration of drug exposure was defined here as the time between the first dose of study drug and endpoint LP. Percent change from baseline for biomarkers are reported in the text as medians. Differences between groups treated with drug or placebo were assessed with two-tailed unpaired Student's t tests at α = 0.05. Linear regression analyses assessing the relationship between each biomarker were performed. No covariates were included in the regression analyses. All statistical tests in this analysis were conducted without adjusting for multiple comparisons so reported statistical significance is considered nominal.

CSF data values that were both two standard deviations above or below the mean and visually deviated from correlations from other measured biomarkers were considered outliers and excluded from the analysis. The biomarker correlations used for the initial outlier screen were the documented correlations between tTau and pTau proteoforms [25,26] as well as Aβ_1–40_ and Aβ_1–42_ [27]. Supplemental Fig. 1 and Supplemental Table 4 illustrate the outlier selection process. No outliers were identified in the plasma analyses.

## 3 Results

### 3.1 Samples analyzed and assay performance

A total of 111 CSF samples were analyzed for Aβ_1–40_, Aβ_1–42_, pTau181, tTau, neurogranin, and NPTX2. For VAMP2, 110 CSF samples were analyzed (one vial had insufficient sample volume for analysis). For CSF pTau217, 98 CSF samples were analyzed (13 samples had insufficient volume for analysis after the measurement of the analytes listed previously). A total of 215 plasma samples were analyzed for Aβ_1–40_, Aβ_1–42_, NfL, GFAP, pTau181, and pTau217. Two participants had outlier data that were removed from the CSF analyses, one in the 60 mg/kg SAD (pTau181 and pTau217) and one in the 60 mg/kg MAD (Aβ_1–40_, Aβ_1–42_). Measured biomarker concentrations across all cohorts in CSF and plasma are shown in Supplemental Table 5. No drug interference was observed in the CSF assay at drug concentrations (50 – 500 pg/mL) spanning CSF exposure levels measured in INTERCEPT-AD; however, because significant drug interference was observed for the plasma Aβ_1–42_ and Aβ_1–40_ assays, these data are not reported.

### 3.2 CSF biomarkers

**Fig. 2:**
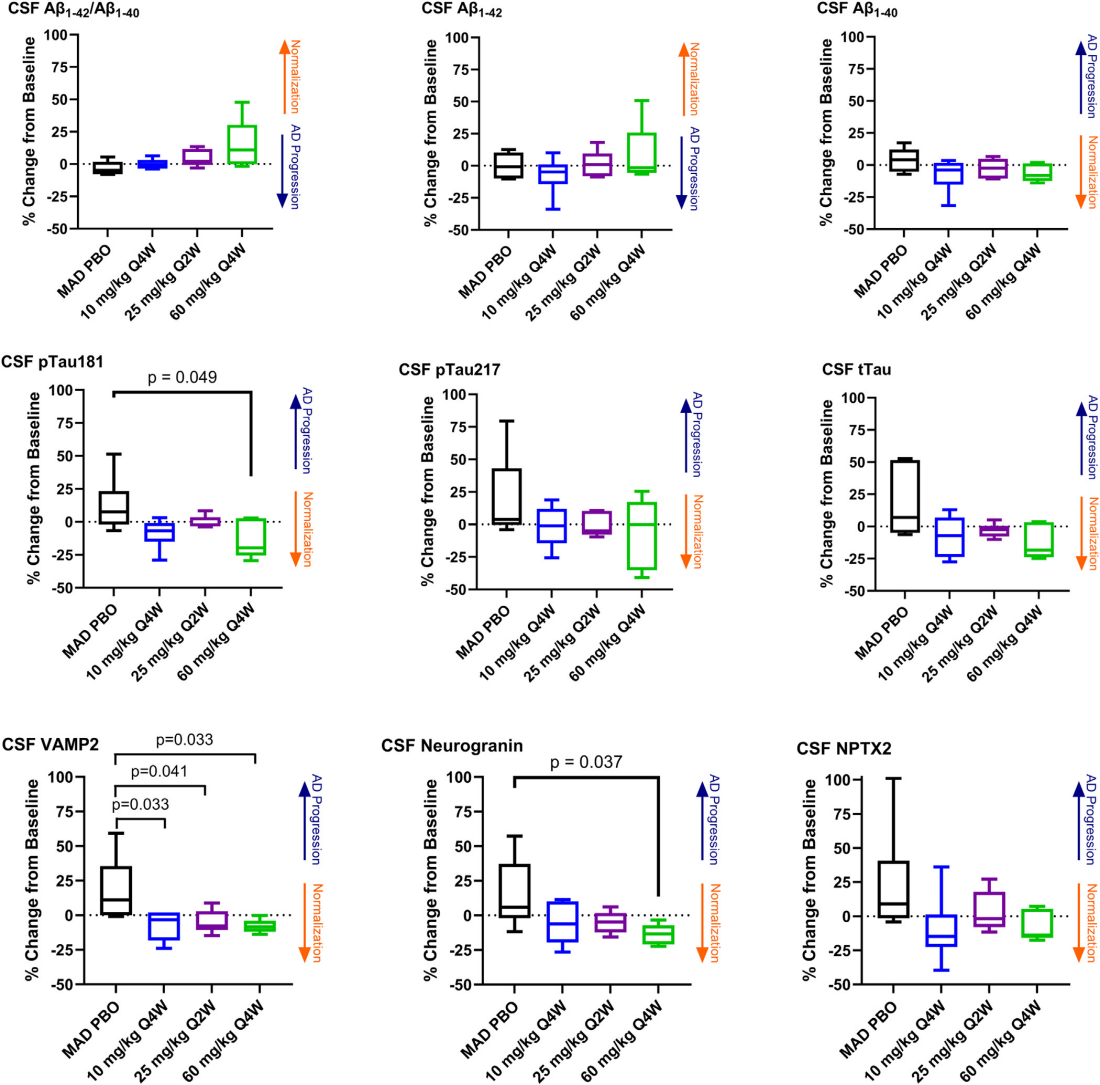
Changes in Biomarker Cerebrospinal Fluid Concentrations After Three Sabirnetug Doses. The graphs show percent changes in CSF biomarker concentrations from baseline to endpoint lumbar puncture for participants in MAD cohorts 5, 6, and 7 who received placebo or sabirnetug. The endpoint lumbar puncture occurred on Day 70, 63, and 35, respectively, for cohorts 5, 6, and 7. Box and whisker plots present median (line), the interquartile range (boxes aka hinges), and minimum and maximum (whiskers). Aβ, amyloid-β; CSF, cerebrospinal fluid; MAD, multiple ascending dose; NPTX2, neuronal pentraxin 2; PBO, placebo; pTau181 or 217, tau phosphorylated at site 181 or 217; Q2W, every 2 weeks; Q4W, every 4 weeks; tTau, total tau protein; VAMP2, vesicle-associated membrane protein 2.

**Fig. 3:**
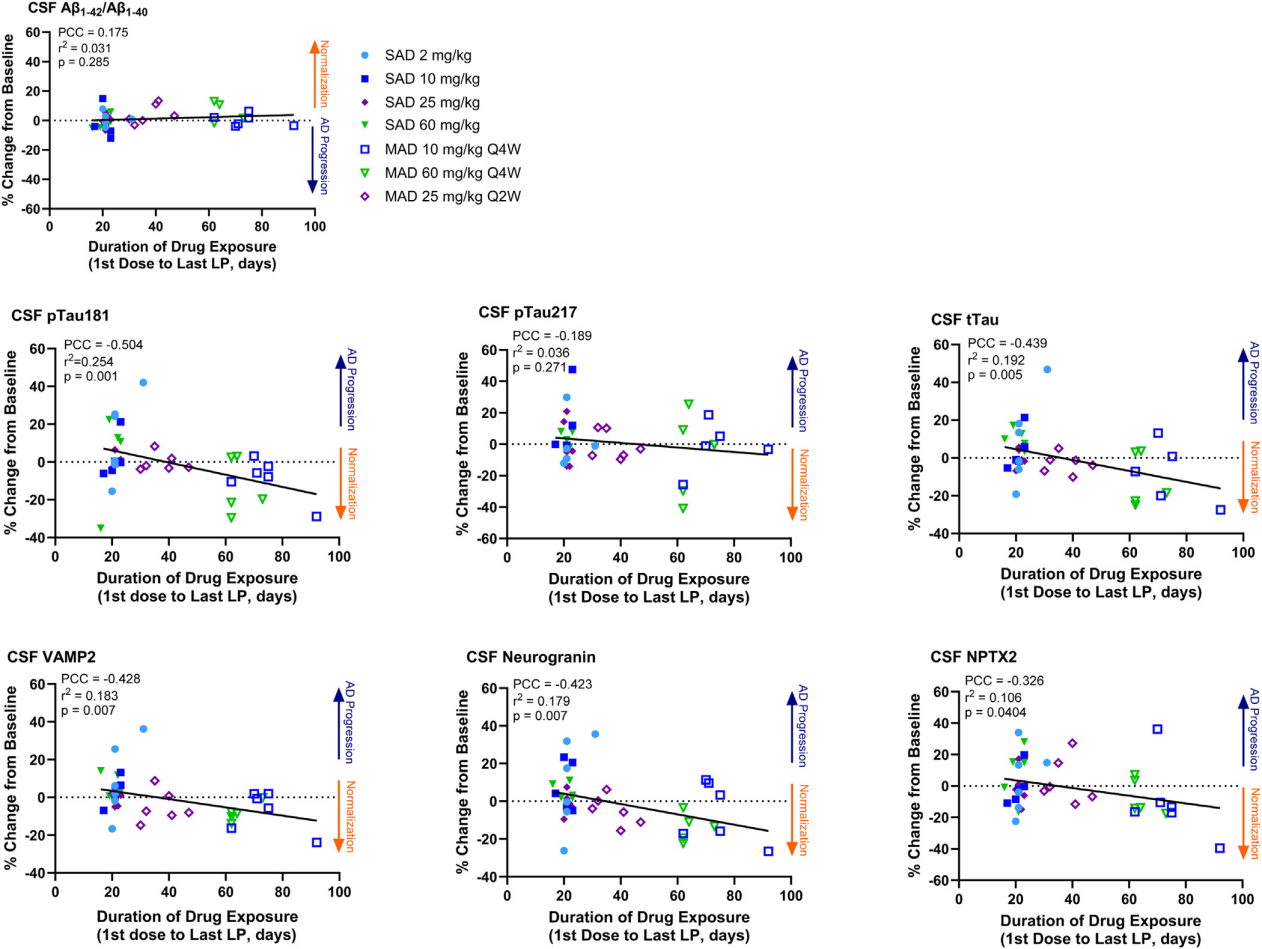
Correlations Between Sabirnetug Dose and Treatment Duration and Biomarker Concentrations in Cerebrospinal Fluid. Correlation plots present data points from individual study participants with lines from linear regressions. Aβ, amyloid-β; CSF, cerebrospinal fluid; MAD, multiple ascending dose; NPTX2, neuronal pentraxin 2; pTau181 or 217, tau phosphorylated at site 181 or 217; Q2W, every 2 weeks; Q4W, every 4 weeks; SAD, single ascending dose; tTau, total tau protein; VAMP2, vesicle-associated membrane protein 2.

**Fig. 4:**
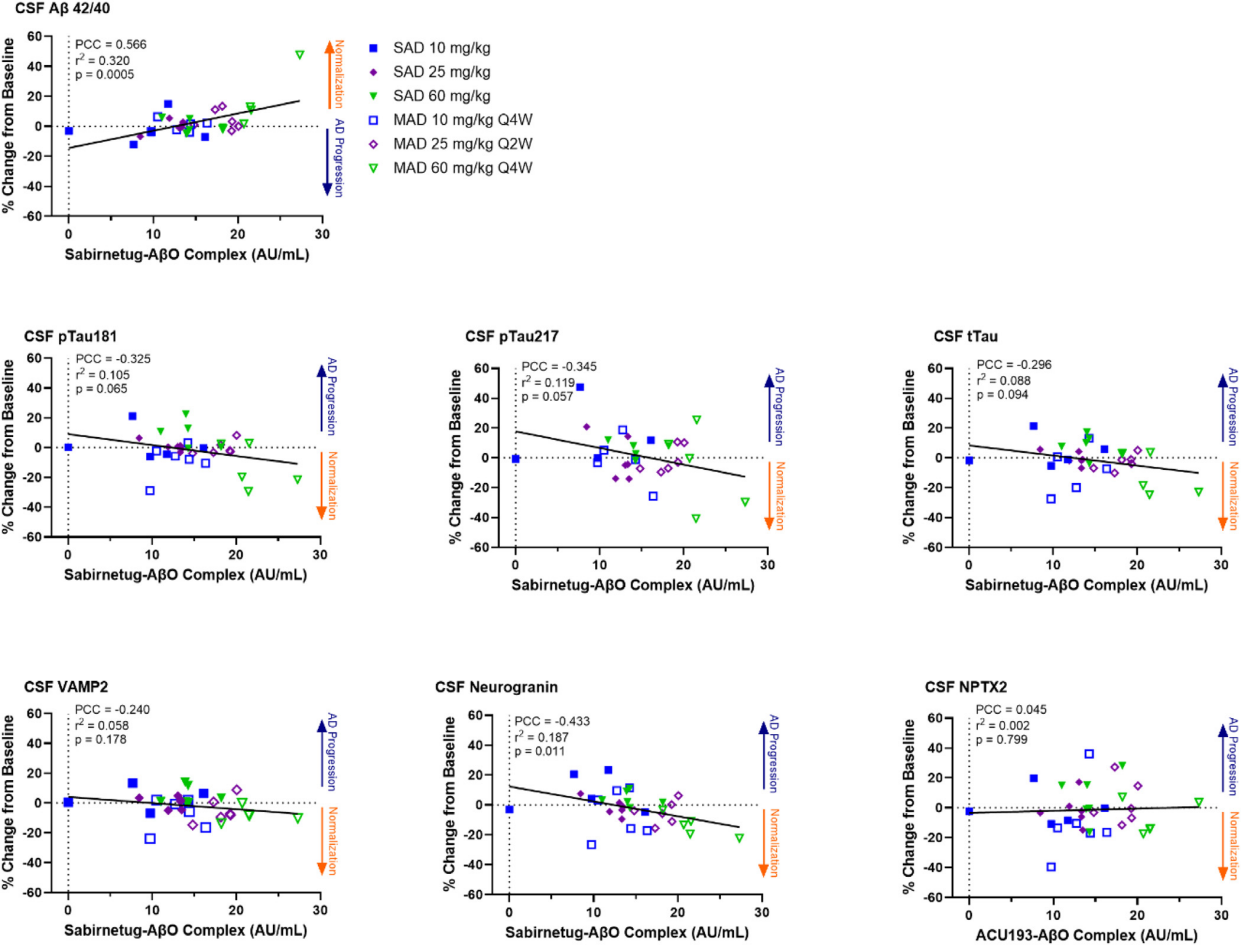
Correlations between Sabirnetug Target Engagement and Biomarker Concentrations in Cerebrospinal Fluid. Correlation plots present data points from individual study participants with lines from linear regressions. Aβ, amyloid-β; AβO, amyloid-β oligomer; CSF, cerebrospinal fluid; MAD, multiple ascending dose; NPTX2, neuronal pentraxin 2; pTau181 or 217, tau phosphorylated at site 181 or 217; Q2W, every 2 weeks; Q4W, every 4 weeks; SAD, single ascending dose; tTau, total tau protein; VAMP2, vesicle-associated membrane protein 2.

The CSF Aβ_1–42_/Aβ_1–40_ ratio in sabirnetug-treated participants in the MAD cohorts increased after three doses (Fig. 2), with a trend towards increasing Aβ_1–42_/Aβ_1–40_ ratio with increasing doses in these cohorts. Accordingly, the largest median increase in the ratio from baseline was observed at the 60 mg/kg Q4W dose (10.9 %, Fig. 2 and Supplemental Tables 6 and 7). The change in the ratio was numerically driven by an increase in Aβ_1–42_ levels and a slight decrease in Aβ_1–40_ levels (Fig. 2). Changes in Aβ_1–42_/Aβ_1–40_ did not significantly correlate with duration of drug exposure (Fig. 3 and Supplemental Table 8). However, a positive correlation was observed between the increase in Aβ_1–42_/Aβ_1–40_ and sabirnetug target engagement (i.e., the sabirnetug-AβO complex in CSF) (*p* = 0.0005; R^2^ = 0.320; Fig. 4 and Supplemental Table 9). As described previously, sabirnetug target engagement was dose- and exposure-dependent in INTERCEPT-AD starting at the 10 mg/kg single dose and approaching saturation at the 25 mg/kg Q2W dose (E_max_ = 22.71 AU/mL sabirnetug-AβO complex) [11].

For CSF pTau181, a nominally significant median change from baseline of −19.6 %, (*p* = 0.049, Fig. 2 and Supplemental Tables 6 and 7) was measured in the 60 mg/kg Q4W sabirnetug-treated participants versus the placebo-treated participants, which showed an increase. The decrease of CSF pTau181 following sabirnetug treatment was significantly correlated with increasing duration of drug exposure (*p* = 0.001, R^2^ = 0.254, Fig. 3 and Supplemental Table 8) and trended toward correlation with target engagement (*p* = 0.065, R^2^ = 0.105, Fig. 4 and Supplemental Table 9) but not decrease in amyloid plaque (*p* = 0.288, R^2^ = 0.031, Supplemental Fig. 3 and Supplemental Table 10). pTau217 levels in sabirnetug-treated MAD cohorts changed less from baseline than the placebo group (Fig. 2). The percent change in pTau217 following sabirnetug treatment across relevant cohorts trended toward correlation with target engagement (p = 0.057, R^2^ = 0.119, Fig. 4 and Supplemental Table 9) and decrease in amyloid plaque (p=0.044, R^2^ = 0.118, Supplemental Fig. 3 and Supplemental Table 10) but not duration of drug exposure (p = 0.271, R^2^ = 0.036; Fig. 3 and Supplemental Table 8). Finally, sabirnetug-treated participants showed a numeric median decrease in tTau in the MAD cohorts, which was greatest in the 60 mg/kg Q4W cohort (−18.5 %, *p* = 0.06, Fig. 2 and Supplemental Tables 6 and 7). This decrease was correlated with increasing duration of drug exposure (*p* = 0.005, R^2^ = 0.192, Fig. 3 and Supplemental Table 8) and trended toward correlation with target engagement (*p* = 0.09, R^2^ = 0.088, Fig. 4 and Supplemental Table 9).

CSF levels of the synaptic biomarkers VAMP2 and neurogranin decreased compared to baseline in the MAD cohorts. NPTX2 showed less change from baseline in sabirnetug-treated participants in the MAD than in the placebo group. By contrast, levels of these three synaptic biomarkers increased from baseline following placebo treatment (Fig. 2 and Supplemental Table 6). The decrease of VAMP2 reached nominal statistical significance versus placebo at all doses (−3.2 %, *p* = 0.033; −7.7 %, *p* = 0.041; −8.4 %, *p* = 0.033 for 10 mg/kg Q4W, 25 mg/kg Q2W, 60 mg/kg Q4W, respectively) and the decrease of neurogranin reached nominal statistical significance versus placebo in the 60 mg/kg Q4W cohort (−13.3 %, *p* = 0.037, Fig. 2 and Supplemental Table 7). The decrease of VAMP2 and neurogranin following sabirnetug treatment was significantly correlated with increasing duration of drug exposure (*p* = 0.007 for both analytes, R^2^ = 0.183 for VAMP2 and 0.179 for neurogranin, Fig. 3 and Supplemental Table 8) and the decrease of neurogranin was significantly correlated with target engagement (*p* = 0.01, R^2^ = 0.187, Fig. 4 and Supplemental Table 9).

No statistically significant differences in baseline to endpoint changes in CSF biomarker concentrations were observed among participants in the SAD cohorts (Supplemental Fig. 2).

### 3.3 Plasma biomarkers

**Fig. 5:**
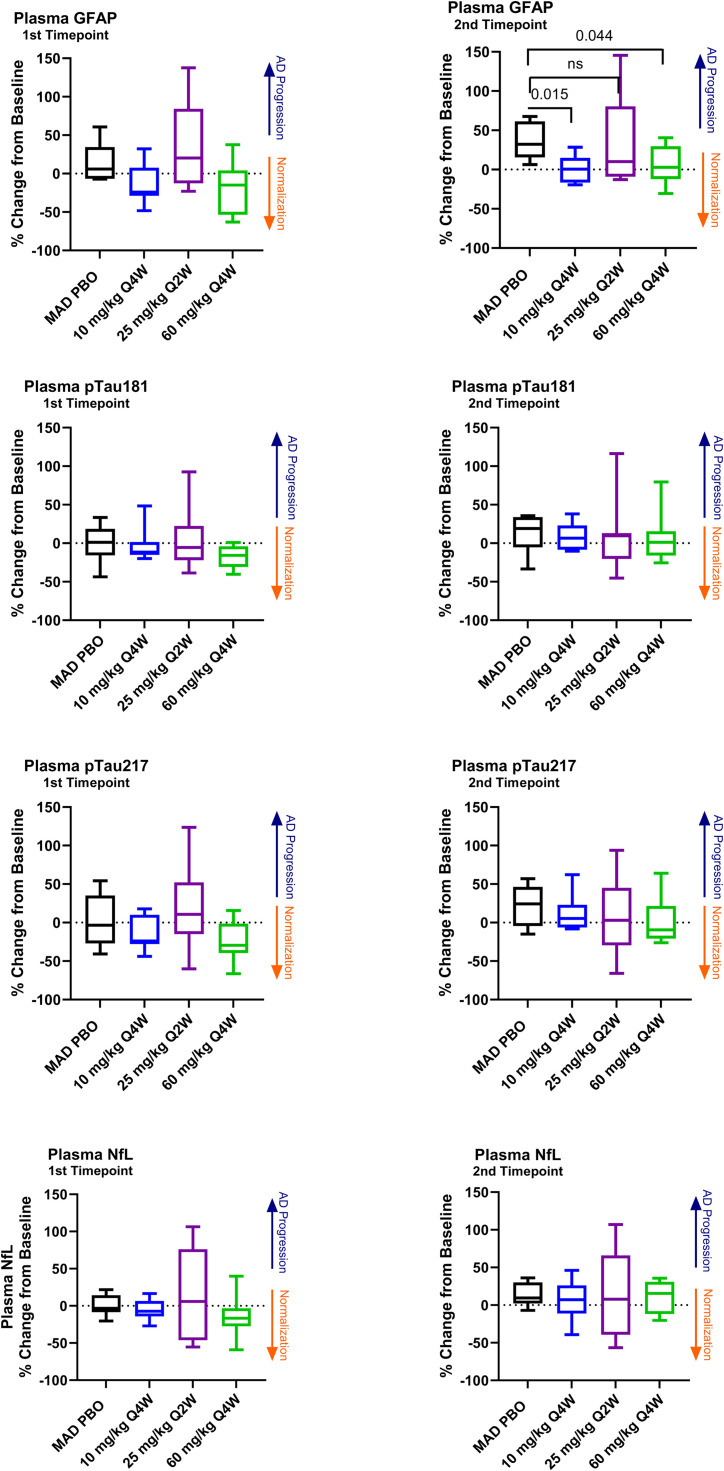
Changes in Plasma Biomarker Concentrations After Three Sabirnetug Doses (1st Timepoint) and After Sabirnetug Washout (2nd Timepoint). The graphs show percent changes in plasma biomarker concentrations from baseline to plasma sampling for participants in MAD cohorts 5, 6, and 7 who received placebo or sabirnetug. Plasma samples for 1st Timepoint were collected at 14, 7, and 7 days, respectively, after the last dose in cohorts 5, 6, and 7. Plasma samples for 2nd Timepoint were collected at 140, 70, and 70 days, respectively, after the last dose in cohorts 5, 6, and 7. Box and whisker plots present median (line), the interquartile range (boxes aka hinges), and minimum and maximum (whiskers). GFAP, glial fibrillary acidic protein; pTau181 or 217, tau phosphorylated at site 181 or 217; NfL, neurofilament light; ns, non-significant (*p* > 0.05); Q2W, every 2 weeks; Q4W, every 4 weeks; tTau, total tau protein.

Plasma concentrations of GFAP, pTau181, pTau217, and NfL decreased numerically from baseline in the 10 mg/kg and 60 mg/kg Q4W MAD cohorts at the first plasma sampling timepoint, after three sabirnetug doses (Fig. 5 and Supplemental Table 11). At the final sampling timepoint for those two cohorts (70–140 days after the third sabirnetug dose), concentrations of these biomarkers increased towards baseline levels (Fig. 5) but remained lower than placebo levels at the end of the study. This difference was nominally statistically significant in the Q4W cohorts for GFAP (p = 0.015, 10 mg/kg Q4W; *p* = 0.044, 60 mg/kg Q4W, Fig. 5, Supplemental Table 12).

For the SAD cohorts, plasma samples for biomarker testing were collected at baseline and on days 4, 21, and 140 after a single infusion of sabirnetug. No discernible sabirnetug-dependent changes in plasma biomarkers were observed over these three timepoints (Supplemental Table 11).

## 4 Discussion

In INTERCEPT-AD, three administrations of sabirnetug resulted in lowering CSF levels of biomarkers that are elevated in AD, pTau181 and tTau, as well as the synaptic injury markers VAMP2 and neurogranin. Additionally, the Aβ_1–42_/Aβ_1–40_ ratio trended towards an increase following sabirnetug treatment. CSF NPTX2 and pTau217 had less change from baseline levels after three administrations of sabirnetug than after placebo administration. In the MAD placebo groups, the change in CSF biomarker concentrations during the study were consistent with the natural course of the disease pathology: decreases were seen in the Aβ_1–42_/Aβ_1–40_ ratio while increases were seen in the other CSF biomarkers examined. The sabirnetug-dependent changes in CSF biomarkers were generally correlated with duration of sabirnetug exposure and also generally trended towards correlation with sabirnetug target engagement with AβOs in CSF. In line with published findings, CSF pTau217 correlated with change in amyloid plaque but CSF pTau181 did not [28]. Plasma concentrations of GFAP, pTau181, pTau217, and NfL decreased numerically when sampled within 6 weeks of the last of three administrations of 10 mg/kg or 60 mg/kg Q4W sabirnetug. Taken together, these data support a rapid downstream PD effect after three administrations of sabirnetug. This includes a notable effect on synaptic biomarkers, which is consistent with the proposed mechanism of sabirnetug targeting AβOs and the binding of AβOs to synapses in nonclinical studies [4,12,29].

The synaptic marker VAMP2 was most sensitive to sabirnetug treatment, with nominally significant decreases in all three MAD cohorts. VAMP2 is part of the soluble N-ethylmaleimide-sensitive factor attached protein receptor (SNARE) complex, involved in the synaptic neurotransmitter release mechanism. Nonclinical studies have identified linkages between AβOs and VAMP2. AβOs have been shown to disrupt SNARE complex formation in transgenic mice through direct interaction with syntaxin 1a, which directly binds VAMP2 in the complex [30]. Furthermore, AβO addition to human iPSC-derived neuronal cultures has been shown to increase VAMP2 protein expression levels [31]. However, studies with larger sample sizes are needed to confirm whether VAMP2 or other synaptic biomarkers are particularly sensitive to sabirnetug treatment. For example, synaptosomal-associated protein-25 (SNAP25) may be of particular interest as it is also part of the SNARE complex [32,33].

Comparatively more robust biomarker data were collected in INTERCEPT-AD than in phase 1 trials of other amyloid or Aβ-targeting monoclonal antibody treatments for AD, including lecanemab and donanemab, due to the increased availability of novel and robust biomarker assays. No biomarker data were obtained in the report of the phase 1b trial of donanemab [34] or the phase 1 trials of gantenerumab [35]. In the phase 1 lecanemab clinical trial, CSF concentrations of Aβ_1–42_, pTau181, and tTau were measured, but no effect was observed compared to placebo for these biomarkers after four doses (0.3, 1, 3, or 10 mg/kg lecanemab) administered Q4W [36]. In contrast, a nominally significant difference from placebo for pTau181 after 3 doses of 60 mg/kg sabirnetug Q4W was observed in INTERCEPT-AD.

Some phase 2 and 3 trials of amyloid or Aβ-targeting monoclonal antibody treatments for AD have reported CSF neurogranin data. For example, the phase 2/3 DIAN-TU-001 trial testing gantenerumab and solanezumab in dominantly inherited AD showed that the Aβ-aggregate selective antibody gantenerumab [37], but not the Aβ monomeric selective antibody solanezumab [38] significantly decreased CSF neurogranin levels after 4 years of treatment [39]. Treatment with the protofibril-selective antibody lecanemab (10 mg/kg) significantly decreased CSF neurogranin levels after 12 months of treatment in a phase 2b trial [40]. However, neurogranin levels in the lecanemab treated groups increased from 12 to 18 months of treatment to the extent that there was no longer a significant difference from the placebo group at 18 months. In the phase 3 CLARITY trial, lecanemab treatment (10 mg/kg) numerically, but not significantly, decreased CSF neurogranin levels after 12 and 18 months of treatment [41]. These data support the hypothesis that changes in synaptic markers such as neurogranin might be an early indicator of reduced synaptotoxic effects of AβOs and protofibrils following treatment. A larger sample size is needed to determine if the decrease of neurogranin after only three doses of sabirnetug is reproducible and if sabirnetug's AβO selectivity leads to a particularly strong effect on synaptic injury markers.

Small molecules designed to target or modulate Aβ for treatment of AD have also been reported to have a treatment effect on AD and synaptic markers. For example, a phase 2 study of ALZ-801, a small molecule possibly inhibiting Aβ oligomer formation, found that 2 years of treatment with ALZ-801 slowed the decrease of CSF Aβ42, and increased the plasma Aβ_1-42_/Aβ_1-40_ ratio, compared with placebo [42]. This contrasts with the results of this shorter INTERCEPT-AD study, where 3 months of sabirnetug treatment resulted in the CSF Aβ_1-42_/Aβ_1-40_ ratio and CSF Aβ_1-42_ alone trending upwards.

In addition, in the phase 2 SPARC and SHINE trials of CT1812, a modulator of the sigma-2 receptor that prevents and displaces AβOs from binding neuronal synaptic receptors, CT1812 treatment altered a number of synaptic proteins vs. placebo, including synaptotagmin-7, NRXN1, NRXN2, and integrin beta-2 [43]. Although these trials are not directly comparable with INTERCEPT-AD due to differences in sample size, treatment duration, and synaptic markers measured, these data together indicate the utility of Aβ and synaptic markers for monitoring the efficacy of AβO-targeting therapeutics.

Limitations of the INTERCEPT-AD study include the relatively small number of participants and short duration of sabirnetug treatment. In addition, no adjustments for multiple comparisons were made due to the exploratory nature of the analyses. While there may be an increased risk of Type I error, or false positives, this study provides preliminary and broader insights into the effects of sabirnetug on CSF and plasma biomarkers related to AD. Longer-term treatment of a larger participant population in the ongoing phase 2 ALTITUDE-AD study (NCT06335173) of sabirnetug will provide further data on the effects of sabirnetug on these biomarkers, including any correlation with clinical response and with reduction in amyloid plaque as assessed by amyloid PET. Strengths of this study include the comparatively large panel of biomarkers examined in this phase 1 study in participants with early symptomatic AD and the consistent effects of sabirnetug treatment on several biomarkers.

## 5 Conclusions

Following three administrations of sabirnetug, the changes from baseline concentrations of several of the examined CSF and plasma biomarkers of AD differed from placebo in a manner that may indicate beneficial effects of sabirnetug on neurodegeneration. The magnitude of the CSF biomarker response to sabirnetug was generally found to increase with increasing dose, duration of drug exposure, and central drug exposure with increasing target engagement. Combined with the knowledge that sabirnetug reaches the central compartment and engages its intended AβO target in CSF [11], these biomarker results are consistent with sabirnetug administration having beneficial effects on downstream PD related to AD pathophysiology. Long-term changes in clinical cognitive outcomes, biomarkers, and safety will be evaluated in the ongoing, placebo-controlled phase 2 ALTITUDE-AD study of sabirnetug (NCT06335173).

## CRediT authorship contribution statement

**Erika N. Cline:** Writing – original draft, Visualization, Formal analysis, Conceptualization. **Daniel Antwi-Berko:** Writing – review & editing, Validation, Investigation. **Karen Sundell:** Writing – original draft, Visualization. **Elizabeth Johnson:** Writing – review & editing, Visualization, Formal analysis. **Maddelyn Hyland:** Writing – review & editing, Formal analysis. **Hao Zhang:** Writing – review & editing, Methodology. **Hugo Vanderstichele:** Writing – review & editing, Methodology, Conceptualization. **June Kaplow:** Writing – review & editing, Methodology, Conceptualization. **Robert A. Dean:** Writing – review & editing, Methodology, Conceptualization. **Erik Stoops:** Writing – review & editing, Methodology, Investigation. **Eugeen Vanmechelen:** Writing – review & editing, Methodology, Investigation. **Marleen J.A. Koel-Simmelink:** Writing – review & editing, Project administration, Methodology. **Charlotte E. Teunissen:** Writing – review & editing, Supervision, Methodology. **Gopalan Sethuraman:** Writing – review & editing, Formal analysis. **Todd Feaster:** Writing – review & editing, Methodology. **Eric Siemers:** Writing – review & editing, Methodology, Conceptualization. **Jasna Jerecic:** Writing – review & editing, Methodology, Conceptualization.

## Declaration of competing interest

The authors declare the following financial interests/personal relationships which may be considered as potential competing interests:

Erika N. Cline, Karen Sundell, Elizabeth Johnson, Maddelyn Hyland, Hao Zhang, Gopalan Sethuraman, Todd Feaster, Eric Siemers, and Jasna Jerecic are employees of, and minor stockholders in, Acumen Pharmaceuticals, Inc.

Hugo Vanderstichele, June Kaplow, and Robert A. Dean are paid consultants of Acumen Pharmaceuticals, Inc

Eugeen Vanmechelen and Erik Stoops are employees of ADx NeuroSciences, which provided services under contract to Acumen Pharmaceuticals, Inc.

Charlotte E. Teunissen received grants of research funding from the European Commission, the European Partnership on Metrology, The Duch Research Council, The Alzheimer's Drug Discovery Foundation, The Selfridges Group Foundation, Alzheimer Netherlands, Topsector Life Sciences & Health, Acumen Pharmaceuticals, ADX Neurosciences, AC Immune, Alamar, Aribio, Axon-Neurosciences, Beckman-Coulter, BioConnect, Bioorchestra, Brainstorm Therapeutics, Celgene, Cognition Therapy, EIP Pharma, Eisai, Eli Lilly and Company, Fijirebio, Instant Nano Biosciences, Novo Nordisk, Olink, PeopleBio, Quanterix, Roche, Toyama, and Vivoryon. She received consulting and or speaking fees from Aribio, Biogen, Beckman-Coulter, Cognition Therapy, Eli Lilly and Company, Merck, Novo Nordisk, Olink, Roche, and Veravas.

Daniel Antwi-Berko and Marleen J.A. Koel-Simmelink declare no conflict of interests.

## Appendix Supplementary materials

Image, application 1
