# Erratum to “Biofluid biomarker changes following treatment with sabirnetug (ACU193) in INTERCEPT-AD, a phase 1 trial in early Alzheimer's disease” [J Prev Alzheimers Dis 2025;12(4): 100082]

**DOI:** 10.1016/j.tjpad.2025.100217

**Published:** 2025-06-06

**Authors:** Erika N. Cline, Daniel Antwi-Berko, Karen Sundell, Elizabeth Johnson, Maddelyn Hyland, Hao Zhang, Hugo Vanderstichele, June Kaplow, Robert A. Dean, Erik Stoops, Eugeen Vanmechelen, Marleen J.A. Koel-Simmelink, Charlotte E. Teunissen, Gopalan Sethuraman, Todd Feaster, Eric Siemers, Jasna Jerecic

**Affiliations:** a Acumen Pharmaceuticals, Inc, 1210-1220 Washington St., Suite 210, Newton, MA 02465, USA; b Neurochemistry Laboratory, Department of Laboratory Medicine, Amsterdam UMC, Amsterdam, the Netherlands; c ADx NeuroSciences, Technologiepark 6, Gent, Belgium

In this article the funding section is included in the Acknowledgements, in the second paragraph, as shown below (in bold):

We thank Jeroen Vanbrabant, Charlotte Lambrechts and Maxime Van Loo for their technical expertise in preparation of assay reagents and sample analysis for pTau217. Prototype VAMP2 ELISA was prepared by Shreyasee Das. Medical writing assistance was provided by Jennie G. Jacobson of Jacobson Medical Writing, Inc. **and was funded by Acumen Pharmaceuticals.**

Additionally, the authors have provided the following paragraph which details the funding for the study:

## Funding

This study was funded by Acumen Pharmaceuticals, Inc.

*Role of the sponsor:* Employees of Acumen Pharmaceuticals designed and conducted the study, in collaboration with investigators, including data collection and analysis. Employees of Acumen Pharmaceuticals participated as authors on this paper and drafted sections of the manuscript. Employees of Acumen Pharmaceuticals also reviewed the manuscript and provided comments for consideration by the authors.

The Publisher would like to apologize for any inconvenience caused.

