# Supplementary material for: Biofluid biomarker changes following treatment with sabirnetug (ACU193) in INTERCEPT-AD, a phase 1 trial in early Alzheimer's disease
Source: J Prev Alzheimers Dis. 2025 Feb 17;12(4):100082. doi: 10.1016/j.tjpad.2025.100082 (PMC12184040; doi:10.1016/j.tjpad.2025.100082)
Supplement: Supplementary file 1 [file mmc1.docx]

Supplemental Materials

Biofluid biomarker changes following treatment with sabirnetug (ACU193) in INTERCEPT-AD, a phase 1 trial in early Alzheimer’s disease

Erika N. Cline*,^1^ Daniel Antwi-Berko*,^2^ Karen Sundell,^1^ Elizabeth Johnson,^1^ Maddelyn Hyland,^1^ Hao Zhang,^1^ Hugo Vanderstichele,^1^ June Kaplow,^1^ Robert A. Dean,^1^ Erik Stoops,^3^ Eugeen Vanmechelen,^3^ Marleen J.A. Koel-Simmelink,^2^ Charlotte [E. Teunissen](mailto:E.%20Teunissen@amsterdamumc.nl),^2^ Gopalan Sethuraman,^1^ Todd Feaster,^1^ Eric Siemers,^1^ Jasna Jerecic^1^

1. Acumen Pharmaceuticals, Inc, Newton, MA, USA
2. Neurochemistry Laboratory, Department of Laboratory Medicine, Amsterdam UMC, Amsterdam, The Netherlands
3. ADx NeuroSciences, Technologiepark 6, Gent, Belgium

* Authors contributed equally to this work

Supplemental Table 1. Abbreviated Study Schedule: Dose Administration Versus Cerebrospinal Fluid and Plasma Collection

|  | **SAD Cohorts 1-4** | | | | | | |
| --- | --- | --- | --- | --- | --- | --- | --- |
| **Visit** | **2** | **3** | **6** | **8** | **9** | **11** | |
| **Day (Nominal)** | **0** | **1** | **4** | **21** | **42** | **140** | |
| CSF | X |  |  | X |  |  | |
| Plasma Biomarkers | X |  | X^1^ |  | X^2^ | X^3^ | |
| Dose |  | X |  |  |  |  | |
|  | **MAD Cohort 5** | | | | | | |
| **Visit** | **2** | **4** | **5** | | **6** | **7** | |
| **Day (Nominal)** | **0** | **28** | **56** | | **70** | **196** | |
| CSF | X |  |  | | X |  | |
| Plasma Biomarkers | X |  |  | | X^1^ | X^2^ | |
| Dose | X | X | X | |  |  | |
|  | **MAD Cohort 6** | | | | | | |
| **Visit** | **2** | **4** | **5** | | **6** | **7** | |
| **Day (Nominal)** | **0** | **28** | **56** | | **63** | **126** | |
| CSF | X |  |  | | X |  | |
| Plasma Biomarkers | X |  |  | | X^1^ | X^2^ | |
| Dose | X | X | X | |  |  | |
|  | **MAD Cohort 7** | | | | | | |
| **Visit** | **2** | **4** | **5** | | **6** | **7** | **8** |
| **Day (Nominal)** | **0** | **14** | **28** | | **35** | **70** | **98** |
| CSF | X |  |  | | X |  |  |
| Plasma Biomarkers | X |  |  | |  | X^1^ | X^2^ |
| Dose | X | X | X | |  |  |  |

CSF, cerebrospinal fluid.

^1^Timepoint 1; ^2^Timepoint 2; ^3^Timepoint 3

Supplemental Table 2. Cerebrospinal Fluid Biomarker Assays

| Analyte | Platform  Technology | Vendor | Vendor Code | Capture Antibody | Detection Antibody | Performing Lab |
| --- | --- | --- | --- | --- | --- | --- |
| Aβ_1-42_  ([1](#_ENREF_1), [2](#_ENREF_2)) | Lumipulse | Fujirebio  (single analyte) | FRI38390 | 21F12 (Aβ x-42) | 3D6 (Aβ 1-x) | VUMC |
| Aβ_1-40_  ([1](#_ENREF_1), [2](#_ENREF_2)) | Lumipulse |  | FRI00103 | 2G3 (Aβ  x-40) | 3D6 (Aβ 1-x) |  |
| tTau  ([1](#_ENREF_1), [2](#_ENREF_2)) | Lumipulse |  | FRI00075 | AT120 | BT2 + HT7 |  |
| pTau181  ([1](#_ENREF_1), [2](#_ENREF_2)) | Lumipulse |  | FRI00064 | AT270 | BT2 + HT7 |  |
| Neurogranin  ([3](#_ENREF_3), [4](#_ENREF_4)) | ELISA | EUROIMMUN | EQ 6551-9601-L | ADx 451 | ADx403 |  |
| pTau217  ([5](#_ENREF_5)) | Lumipulse G 1200 | ADx (prototype) | prototype | RD-085 (pTau21) | RD-073 (N-terminal tau) | ADx NeuroSciences |
| VAMP2  ([6](#_ENREF_6)) | ELISA | ADx (prototype) | prototype | ADx 451 | ADx 403 | VUMC |
| Neuropentraxin-2 ([7](#_ENREF_7)) | ELISA | Fujirebio | 80908 | 33F9 (ADx 409) | 22H10 (ADx 410) | VUMC |

Aβ, amyloid-β; pTau181 or 217, tau phosphorylated at site 181 or 217; tTau, total tau; VAMP2, vesicle-associated membrane protein 2

Supplemental Table 3. Plasma Biomarker Assays

| Analyte | Platform  Technology | Vendor | Vendor Code | Capture Antibody | Detection Antibody | Performing Lab |
| --- | --- | --- | --- | --- | --- | --- |
| Aβ_1-42_ ([8](#_ENREF_8)) | SimoA  N4PE  (4-Plex) | Quanterix | 103670 | 21F12 (Aβ x-42) ([9](#_ENREF_9)) | 3D6 (Aβ 1-x) ([9](#_ENREF_9)) | VUMC |
| Aβ_1-40_ ([8](#_ENREF_8)) |  |  |  | 2G3 (Aβ x-40) ([9](#_ENREF_9)) | 3D6 (Aβ 1-x) ([9](#_ENREF_9)) |  |
| GFAP ([8](#_ENREF_8)) |  |  |  | Not reported | Not reported |  |
| NfL ([8](#_ENREF_8)) |  |  |  | Not reported | Not reported |  |
| pTau181 ([10](#_ENREF_10)) | SimoA  (Single) |  | 104111  (of 103714) | Not reported | Not reported |  |
| pTau217 ([11](#_ENREF_11)) | SimoA | AlzPath  (Quanterix) | MAB 231122 | Not reported | Not reported |  |

Aβ, amyloid-β; GFAP, glial fibrillary acidic protein; NfL, neurofilament light; pTau181 or 217, tau phosphorylated at site 181 or 217

**Supplemental Table 4. Outlying Values Excluded from Analysis**

| **Cohort, Time Point** | **Correlations in Which Sample is Outlier** | **Outside of Mean ± 2 SD** | **Analysis Values Removed** |
| --- | --- | --- | --- |
| 60 mg/kg SAD, baseline CSF | tTau vs. pTau217  tTau vs. pTau181  pTau181 vs. pTau217 | pTau181  pTau217 | All pTau181 and pTau217 |
| 60 mg/kg Q4W, baseline CSF | Aβ_1-40_ vs. Aβ_1-42_ | Aβ_1-40_  Aβ_1-42_  Aβ_1-42_/Aβ_1-40_ | All Aβ |

Aβ, amyloid-β; CSF, cerebrospinal fluid; pTau181 or 217, tau phosphorylated at site 181 or 217; SAD, single ascending dose; SD, standard deviation; tTau, total tau.

Supplemental Table 5. Measured Biomarker Concentrations Across all Cohorts and Timepoints in CSF and Plasma

| **CSF Biomarkers** | | | |
| --- | --- | --- | --- |
| **Biomarker** | **Median**  **(5^th^ Percentile, 95^th^ Percentile)**  **pg/mL** | **Assay Intra-run Precision*** | |
|  |  | **CV kit QC samples** | **CV lab QC samples** |
| Aβ_1-40_ | 12610 (6448.5, 18925) | 0.4-2.4% CV | 0.6-1.2% CV |
| Aβ_1-42_ | 609 (290.5, 1178.5) | 0.9-1.1% CV | 2.8-3.7% CV |
| pTau181 | 71.2 (32.2, 161.6) | 0-2.4 %CV | 0.9-1.2% CV |
| tTau | 485 (212.5, 938.5) | 0.1-3.1% CV | 0.1-1.7% CV |
| Neurogranin | 539.6 (212.3, 949.9) | 0.4-3.0% CV | 1.4-3.0% CV |
| NPTX2 | 1001.5 (382.6, 2171) | 6.5-9.2% CV | 7.9-11.6% CV |
| VAMP2 | 518.1 (229.8, 830.2) | 5.5-11% CV | Not available |
| pTau217 | 33.224 (9.236, 104.619) | 0.6-10.0% CV | 0.6-10.0% CV |
| **Plasma Biomarkers** | | | |
| **Biomarker** | **Median**  **(5^th^ Percentile, 95^th^ Percentile)**  **pg/mL** | **Assay Intra-run Precision*** | |
|  |  | **CV kit QC samples** | **CV lab QC samples** |
| NfL | 26.4 (13.3, 57.2) | no kit QC samples | 9.7-10% CV |
| GFAP | 185.3 (59.3, 356.1) | no kit QC samples | 9.5-10.9% CV |
| pTau181 | 33 (14.4, 69.7) | no kit QC samples | 7.6-10.7% CV |
| pTau217 | 0.912 (0.262, 2.234) | 7.0-9.9% CV | 8.3-15.7% CV |

*As determined by reference samples; VUMC was the performing laboratory.

Aβ, amyloid-β; GFAP, glial fibrillary acidic protein; NfL, neurofilament light; NPTX2, neuronal pentraxin 2; pTau181 or 217, tau phosphorylated at site 181 or 217; tTau, total tau; VAMP2, vesicle-associated membrane protein 2.

Supplemental Efficacy

Supplemental Table 6. Summary of CSF Biomarkers Following Single and Multiple Dose Administration of Sabirnetug or Placebo (Cohorts 1-7)

|  | **Single Dose Administration** | | | | | | | | | | | **Multiple Dose Administration** | | | | | | | | | | | | |
| --- | --- | --- | --- | --- | --- | --- | --- | --- | --- | --- | --- | --- | --- | --- | --- | --- | --- | --- | --- | --- | --- | --- | --- | --- |
| **CSF Biomarker** | **Placebo** | | **2 mg/kg** | | **10 mg/kg** | | **25 mg/kg** | | **60 mg/kg** | | | **Placebo** | | | **10 mg/kg Q4W** | | | | **25 mg/kg Q2W** | | | | | **60 mg/kg Q4W** |
|  | | | | | | | | | | | | | | | | | | | | | | | | |
| **Aβ_1-42_/Aβ_1-40_**  % Change from BL | |  | |  | | |  | |  | | |  | | |  | | | |  | | | | |  |
| n | 6 | 6 | | 5 | | | 6 | | 6 | | | 5 | | | 6 | | | | 6 | | | | | 5 |
| Mean | 0.77 | 0.85 | | -2.25 | | | 0.12 | | -0.29 | | | -3.39 | | | 0.10 | | | | 4.32 | | | | | 14.32 |
| Standard Deviation | 4.56 | 4.30 | | 10.22 | | | 4.12 | | 4.73 | | | 5.49 | | | 3.98 | | | | 6.53 | | | | | 19.64 |
| Median | -0.12 | 0.09 | | -4.01 | | | 0.05 | | -1.52 | | | -4.77 | | | -0.29 | | | | 2.16 | | | | | 10.88 |
| Min, Max | -2.79, 9.67 | -4.66, 7.98 | | -12.04,14.93 | | | -6.77, 5.45 | | -5.02, 5.87 | | | -8.12, 5.47 | | | -3.84, 6.35 | | | | -3.06, 13.42 | | | | | -1.67, 47.68 |
|  | | | | | | | | | | | | | | | | | | | | | | | | |
| **tTau**  % Change from BL | |  | | | |  | |  | |  | | |  | | |  | | | |  | | | |  |
| n | 6 | 6 | | | | 5 | | 6 | | 6 | | | 6 | | | 5 | | | | 6 | | | | 5 |
| Mean | -1.33 | 8.59 | | | | 3.85 | | -0.29 | | 7.88 | | | 17.86 | | | -8.16 | | | | -3.04 | | | | -11.87 |
| Standard Deviation | 9.25 | 23.09 | | | | 10.59 | | 4.55 | | 7.39 | | | 27.46 | | | 16.17 | | | | 5.24 | | | | 14.08 |
| Median | -0.32 | 5.90 | | | | -1.02 | | -1.40 | | 8.79 | | | 7.04 | | | -7.20 | | | | -2.60 | | | | -18.46 |
| Min, Max | -14.72, 11.13 | -19.18, 46.82 | | | | -5.25, 21.37 | | -6.82, 5.62 | | -3.81, 17.23 | | | -6.31, 52.67 | | | -27.48, 13.11 | | | | -10.12, 5.03 | | | | -24.76, 3.65 |
|  | | | | | | | | | | | | | | | | | | | | | | | | |
| **pTau181**  % Change from BL | |  | |  | | |  | |  | | |  | | |  | | |  | | | | |  | |
| n | 6 | 6 | | 5 | | | 6 | | 5 | | | 6 | | | 6 | | | 6 | | | | 5 | | |
| Mean | -0.00 | 12.54 | | 2.15 | | | 0.83 | | 9.18 | | | 12.22 | | | -8.68 | | | -0.28 | | | | -13.03 | | |
| Standard Deviation | 7.10 | 21.37 | | 10.98 | | | 3.19 | | 9.51 | | | 20.72 | | | 10.98 | | | 4.65 | | | | 14.84 | | |
| Median | 1.38 | 12.40 | | -0.23 | | | 0.60 | | 10.71 | | | 7.60 | | | -6.82 | | | -2.36 | | | | -19.63 | | |
| Min, Max | -11.99, 8.32 | -15.49, 42.01 | | -6.01, 21.19 | | | -3.13, 6.49 | | -0.47, 22.42 | | | -6.69, 51.27 | | | -28.88, 3.20 | | | -3.83, 8.26 | | | | -29.48, 2.95 | | |
|  | | | | | | | | | | | | | | | | | | | | | | | | |
| **pTau217**  % Change from BL | |  | |  | | |  | |  | | |  | | |  | | | |  | | | | |  |
| n | 6 | 5 | | 4 | | | 6 | | 5 | | | 5 | | | 5 | | | | 6 | | | | | 5 |
| Mean | -2.93 | 0.98 | | 14.65 | | | -0.32 | | 5.77 | | | 17.86 | | | -1.22 | | | | -0.96 | | | | | -7.19 |
| Standard Deviation | 11.44 | 16.75 | | 22.62 | | | 14.62 | | 5.35 | | | 34.64 | | | 16.14 | | | | 9.05 | | | | | 27.46 |
| Median | -8.37 | -2.75 | | 5.93 | | | -4.62 | | 7.95 | | | 3.95 | | | -1.06 | | | | -4.93 | | | | | -0.25 |
| Min, Max | -11.86, 13.95 | -12.05, 29.84 | | -0.73, 47.45 | | | -14.00, 20.84 | | -1.81, 11.95 | | | -4.11, 79.40 | | | -25.68, 18.77 | | | | -9.54, 10.65 | | | | | -40.78, 25.48 |
|  | | | | | | | | | | | | | | | | | | | | | | | | |
| **Neurogranin**  % Change from BL | |  | |  | | |  | |  | | |  | | |  | | |  | | | |  | | |
| n | 6 | 6 | | 5 | | | 6 | | 6 | | | 6 | | | 6 | | | 6 | | | | 5 | | |
| Mean | -4.30 | 8.84 | | 8.03 | | | -1.59 | | 4.17 | | | 14.84 | | | -5.85 | | | -4.96 | | | | -13.89 | | |
| Standard Deviation | 10.03 | 23.83 | | 13.17 | | | 5.75 | | 4.91 | | | 25.07 | | | 15.97 | | | 7.80 | | | | 7.44 | | |
| Median | -4.20 | 8.58 | | 4.21 | | | -2.29 | | 2.52 | | | 5.89 | | | -6.21 | | | -4.80 | | | | -13.33 | | |
| Min, Max | -21.79, 5.64 | -26.18, 35.62 | | -4.89, 23.34 | | | -9.42, 7.53 | | -1.95, 11.01 | | | -11.87, 57.28 | | | -26.52, 11.41 | | | -15.57, 6.25 | | | | -22.29, -3.37 | | |
|  | | | | | | | | | | | | | | | | | | | | | | | | |
| **NPTX2**  % Change from BL | |  | |  | | |  | |  | | |  | | |  | | |  | | | |  | | |
| n | 6 | 6 | | 5 | | | 6 | | 6 | | | 6 | | | 6 | | | 6 | | | | 5 | | |
| Mean | 6.59 | 3.69 | | -0.39 | | | -1.39 | | 6.79 | | | 22.51 | | | -10.10 | | | 3.31 | | | | -6.94 | | |
| Standard Deviation | 25.92 | 20.91 | | 12.05 | | | 10.53 | | 15.74 | | | 39.47 | | | 24.91 | | | 14.71 | | | | 11.41 | | |
| Median | -5.02 | 4.77 | | -2.31 | | | -2.80 | | 7.27 | | | 9.09 | | | -14.90 | | | -1.83 | | | | -13.78 | | |
| Min, Max | -10.50, 57.32 | -22.46, 34.04 | | -10.85, 19.73 | | | -14.82, 17.10 | | -16.32, 28.15 | | | -4.16, 101.14 | | | -39.56, 36.12 | | | -11.60, 27.24 | | | | -17.45, 7.14 | | |
|  | | | | | | | | | | | | | | | | | | | | | | | | |
| **VAMP2**  % Change from BL | |  | |  | | |  | |  | |  | | |  | | |  | | | |  | | | |
| n | 6 | 6 | | 4 | | | 6 | | 6 | | 6 | | | 6 | | | 6 | | | | 5 | | | |
| Mean | -4.32 | 8.58 | | 3.33 | | | 0.36 | | 5.18 | | 18.09 | | | -7.13 | | | -4.98 | | | | -8.09 | | | |
| Standard Deviation | 7.39 | 19.24 | | 8.55 | | | 4.17 | | 6.11 | | 22.63 | | | 10.70 | | | 8.39 | | | | 4.98 | | | |
| Median | -2.24 | 3.99 | | 3.47 | | | 1.67 | | 2.26 | | 11.08 | | | -3.18 | | | -7.67 | | | | -8.42 | | | |
| Min, Max | -17.94, 3.79 | -16.60, 36.29 | | -6.86, 13.23 | | | -4.96, 4.89 | | 0.11, 14.04 | | -0.86, 59.28 | | | -23.89, 1.95 | | | -14.68, 8.82 | | | | -13.75, -0.19 | | | |

Aβ, amyloid-β; BL, baseline; NPTX2, neuronal pentraxin 2; NRGN, neurogranin; pTau181 or 217, tau phosphorylated at site 181 or 217; tTau, total tau; Q2W, every 2 weeks; Q4W, every 4 weeks; VAMP2, vesicle-associated membrane protein 2

CSF samples were collected on days 0 and 21 for all SAD cohorts, days 0 and 70 for cohort 5, days 0 and 63 for cohort 6, and days 0 and 35 for cohort 7.

Supplemental Table 7. Unpaired Student's T Test (two-tailed) Statistics for Percent Change in Cerebrospinal Fluid Biomarkers Results

| **CSF** **Biomarker** | **Comparison** **Group** **1** | **Comparison** **Group** **2** | **Mean** **Difference** | **t** **Statistic** | **Degrees** **of** **Freedom** | **p-value** |
| --- | --- | --- | --- | --- | --- | --- |
| Aβ_1−40_ | Sabirnetug 10 mg/kg Q4W | Placebo | -11.0368 | -1.58 | 9 | 0.1488 |
| Aβ_1−40_ | Sabirnetug 25 mg/kg Q2W | Placebo | -6.0977 | -1.19 | 9 | 0.2641 |
| Aβ_1−40_ | Sabirnetug 60 mg/kg Q4W | Placebo | -9.6878 | -1.84 | 8 | 0.1035 |
|  | | | | | | |
| Aβ_1−42_ | Sabirnetug 10 mg/kg Q4W | Placebo | -7.2179 | -0.93 | 9 | 0.3750 |
| Aβ_1−42_ | Sabirnetug 25 mg/kg Q2W | Placebo | 1.6633 | 0.27 | 9 | 0.7911 |
| Aβ_1−42_ | Sabirnetug 60 mg/kg Q4W | Placebo | 7.7829 | 0.66 | 8 | 0.5257 |
|  | | | | | | |
| Aβ_1-42_/Aβ_1-40_  Ratio | Sabirnetug 10 mg/kg Q4W | Placebo | 3.4948 | 1.22 | 9 | 0.2517 |
| Aβ_1-42_/Aβ_1-40_  Ratio | Sabirnetug 25 mg/kg Q2W | Placebo | 7.7136 | 2.09 | 9 | 0.0660 |
| Aβ_1-42_/Aβ_1-40_  Ratio | Sabirnetug 60 mg/kg Q4W | Placebo | 17.7127 | 1.94 | 8 | 0.0880 |
|  | | | | | | |
| tTau | Sabirnetug 10 mg/kg Q4W | Placebo | -26.0187 | -1.86 | 9 | 0.0962 |
| tTau | Sabirnetug 25 mg/kg Q2W | Placebo | -20.8972 | -1.83 | 10 | 0.0970 |
| tTau | Sabirnetug 60 mg/kg Q4W | Placebo | -29.7242 | -2.18 | 9 | 0.0572 |
|  | | | | | | |
| pTau181 | Sabirnetug 10 mg/kg Q4W | Placebo | -20.8921 | -2.18 | 10 | 0.0540 |
| pTau181 | Sabirnetug 25 mg/kg Q2W | Placebo | -12.4925 | -1.44 | 10 | 0.1802 |
| pTau181 | Sabirnetug 60 mg/kg Q4W | Placebo | -25.2464 | -2.27 | 9 | 0.0491 |
|  | | | | | | |
| pTau217 | Sabirnetug 10 mg/kg Q4W | Placebo | -19.0868 | -1.12 | 8 | 0.2964 |
| pTau217 | Sabirnetug 25 mg/kg Q2W | Placebo | -18.8229 | -1.29 | 9 | 0.2285 |
| pTau217 | Sabirnetug 60 mg/kg Q4W | Placebo | -25.0569 | -1.27 | 8 | 0.2406 |
|  | | | | | | |
| NPTX2 | Sabirnetug 10 mg/kg Q4W | Placebo | -32.6079 | -1.71 | 10 | 0.1178 |
| NPTX2 | Sabirnetug 25 mg/kg Q2W | Placebo | -19.1933 | -1.12 | 10 | 0.2905 |
| NPTX2 | Sabirnetug 60 mg/kg Q4W | Placebo | -29.4497 | -1.60 | 9 | 0.1440 |
|  | | | | | | |
| Neurogranin | Sabirnetug 10 mg/kg Q4W | Placebo | -20.6884 | -1.70 | 10 | 0.1190 |
| Neurogranin | Sabirnetug 25 mg/kg Q2W | Placebo | -19.7950 | -1.85 | 10 | 0.0945 |
| Neurogranin | Sabirnetug 60 mg/kg Q4W | Placebo | -28.7237 | -2.45 | 9 | 0.0365 |
|  | | | | | | |
| VAMP2 | Sabirnetug 10 mg/kg Q4W | Placebo | -25.2202 | -2.47 | 10 | 0.0332 |
| VAMP2 | Sabirnetug 25 mg/kg Q2W | Placebo | -23.0720 | -2.34 | 10 | 0.0412 |
| VAMP2 | Sabirnetug 60 mg/kg Q4W | Placebo | -26.1856 | -2.52 | 9 | 0.0330 |

Aβ, amyloid-β; BL, baseline; CSF, cerebrospinal fluid; NPTX2, neuronal pentraxin 2; pTau181 or 217, tau phosphorylated at site 181 or 217; tTau, total tau; Q2W, every 2 weeks; Q4W, every 4 weeks; VAMP2, vesicle-associated membrane protein 2

T-tests were conducted assuming equal variances; mean differences were calculated with the following formula: (Comparison Group 1) - (Comparison Group 2)

CSF samples were collected on days 0 and 21 for all SAD cohorts, days 0 and 70 for cohort 5, days 0 and 63 for cohort 6, and days 0 and 35 for cohort 7.

Supplemental Table 8. Pearson Correlation and Simple Linear Regression Statistics for Duration of Drug Exposure and CSF Biomarkers (Cohorts 1-7)

|  | | **Pearson** **Correlation** | | **Simple** **Linear** **Regression** | | |
| --- | --- | --- | --- | --- | --- | --- |
| **CSF** **Biomarker** | **n** | **Pearson** **Correlation** **Coefficient** | **p-value** | **Slope** | **Y-intercept** | **R-squared** |
| Aβ_1-42_/Aβ_1-40_ Ratio | 39 | 0.1754851 | 0.2852644 | 0.0486781 | -0.665082 | 0.030795 |
| NPTX2 | 40 | -0.325552 | 0.0403743 | -0.244531 | 8.5020692 | 0.1059841 |
| Neurogranin | 40 | -0.423068 | 0.0065301 | -0.273853 | 9.5201046 | 0.1789867 |
| pTau181 | 39 | -0.503889 | 0.001074 | -0.322222 | 12.588542 | 0.2539044 |
| pTau217 | 36 | -0.188561 | 0.2707435 | -0.144643 | 6.6157445 | 0.0355551 |
| tTau | 39 | -0.438555 | 0.0052272 | -0.288514 | 10.370094 | 0.1923307 |
| VAMP2 | 39 | -0.428358 | 0.0065167 | -0.218643 | 7.821705 | 0.1834904 |

Aβ, amyloid-β; CSF, cerebrospinal fluid; NPTX2, neuronal pentraxin 2; pTau181 or 217, tau phosphorylated at site 181 or 217; tTau, total tau; VAMP2, vesicle-associated membrane protein 2

Supplemental Table 9. Pearson Correlation and Simple Linear Regression Statistics for CSF Biomarkers and Sabirnetug-AβO Complex (Cohorts 1-7)

|  | | **Pearson** **Correlation** | | **Simple** **Linear** **Regression** | | |
| --- | --- | --- | --- | --- | --- | --- |
| **CSF**  **Biomarker** | **n** | **Pearson** **Correlation** **Coefficient** | **p-value** | **Slope** | **Y-intercept** | **R-squared** |
| Aβ_1-42_/Aβ_1-40_ Ratio | 34 | 0.5657318 | 0.0004886 | 1.1508968 | -14.41821 | 0.3200525 |
| NPTX2 | 34 | 0.0454269 | 0.7986412 | 0.1438423 | -3.440272 | 0.0020636 |
| Neurogranin | 34 | -0.432631 | 0.0106044 | -0.99671 | 12.35995 | 0.1871693 |
| pTau181 | 33 | -0.324575 | 0.0653436 | -0.729041 | 9.0150209 | 0.1053491 |
| pTau217 | 31 | -0.345142 | 0.0572234 | -1.113692 | 17.760615 | 0.1191229 |
| tTau | 33 | -0.296427 | 0.0939203 | -0.674983 | 8.3194505 | 0.087869 |
| VAMP2 | 33 | -0.24006 | 0.1784193 | -0.410233 | 4.0613546 | 0.0576286 |

Aβ, amyloid-β; BL, baseline; NPTX2, neuronal pentraxin 2; NRGN, neurogranin; pTau181 or 217, tau phosphorylated at site 181 or 217; tTau, total tau; VAMP2, vesicle-associated membrane protein 2

Supplemental Table 10. Pearson Correlation and Simple Linear Regression Statistics for CSF Phosphorylated Tau and Plaque Reduction (Cohorts 1-7)

|  | | **Pearson** **Correlation** | | **Simple** **Linear** **Regression** | | |
| --- | --- | --- | --- | --- | --- | --- |
| **CSF Biomarker** | **n** | **Pearson** **Correlation** **Coefficient** | **p-value** | **Slope** | **Y-intercept** | **R-squared** |
| pTau181 | 38 | 0.1770126 | 0.2877133 | 0.1558919 | 1.8460568 | 0.0313334 |
| pTau217 | 35 | 0.3431235 | 0.0435963 | 0.3485052 | 4.2974133 | 0.1177337 |

CSF, cerebrospinal fluid; pTau181 or 217, tau phosphorylated at site 181 or 217

**Supplemental Table 11. Changes in Plasma Biomarkers**

|  | **Single** **Dose** **Administration** | | | | |  | **Multiple** **Dose** **Administration** | | | |
| --- | --- | --- | --- | --- | --- | --- | --- | --- | --- | --- |
| **Plasma Biomarker/ Timepoint** | **Placebo** | **2**  **mg/kg** | **10**  **mg/kg** | **25**  **mg/kg** | **60**  **mg/kg** | **Plasma Biomarker/ Timepoint** | **Placebo** | **10mg/kg** **Q4W** | **25mg/kg** **Q2W** | **60mg/kg** **Q4W** |
| **pTau181**  % Change from BL | | | | | | **pTau181**  % Change from BL | | | | |
| **Timepoint 1** |  |  |  |  |  | **Timepoint 1** |  |  |  |  |
| n | 7 | 6 | 6 | 6 | 6 | n | 6 | 7 | 8 | 8 |
| Mean | -10.98 | -12.64 | -8.23 | -5.02 | -10.31 | Mean | -0.10 | -1.91 | 5.07 | -17.78 |
| SD | 16.25 | 17.21 | 25.27 | 37.87 | 16.77 | SD | 25.77 | 23.35 | 40.67 | 14.35 |
| Median | -6.56 | -14.14 | -7.50 | -4.64 | -10.98 | Median | 1.11 | -11.60 | -5.59 | -15.83 |
| Min, Max | -34.68,  12.87 | -39.42,  8.06 | -49.50,  24.91 | -47.76,  62.55 | -29.78,  12.92 | Min, Max | -43.58,  33.41 | -19.91,  48.40 | -38.58,  92.65 | -40.22,  0.84 |
| **Timepoint 2** |  |  |  |  |  | **Timepoint 2** |  |  |  |  |
| n | 8 | 6 | 6 | 6 | 6 | n | 6 | 6 | 8 | 8 |
| Mean | -1.14 | -6.27 | 7.14 | -18.38 | -4.46 | Mean | 12.96 | 8.49 | 9.69 | 6.13 |
| SD | 18.48 | 21.33 | 37.25 | 15.46 | 16.65 | SD | 25.52 | 18.00 | 47.97 | 32.87 |
| Median | -2.67 | -7.19 | -7.00 | -10.68 | 3.17 | Median | 19.11 | 6.50 | 9.76 | 1.09 |
| Min, Max | -23.49,  31.35 | -35.53,  18.73 | -21.57,  79.23 | -45.20,  -6.87 | -27.62,  8.89 | Min, Max | -33.53,  35.83 | -10.29,  38.02 | -45.31,  116.25 | -25.51,  79.58 |
| **Timepoint 3** |  |  |  |  |  |  |  |  |  |  |
| n | 8 | 6 | 5 | 5 | 6 |  |  |  |  |  |
| Mean | -0.05 | -16.48 | 18.67 | -7.09 | -2.12 |  |  |  |  |  |
| SD | 11.60 | 21.46 | 19.26 | 19.22 | 13.96 |  |  |  |  |  |
| Median | -3.01 | -8.07 | 12.46 | -1.30 | 3.39 |  |  |  |  |  |
| Min, Max | -11.62,  23.54 | -57.34,  0.67 | 3.09,  49.54 | -36.89,  9.85 | -20.24,  10.13 |  |  |  |  |  |
|  | | | | | |  | | | | |
| **pTau217**  % Change from BL | | | | | | **pTau217**  % Change from BL | | | | |
| **Timepoint 1** |  |  |  |  |  | **Timepoint 1** |  |  |  |  |
| n | 7 | 6 | 6 | 6 | 6 | n | 6 | 7 | 8 | 8 |
| Mean | -18.20 | 1.34 | 2.89 | -8.69 | -12.22 | Mean | 2.19 | -16.05 | 18.93 | -24.28 |
| SD | 25.69 | 26.88 | 32.19 | 28.09 | 18.76 | SD | 34.86 | 22.06 | 55.76 | 26.23 |
| Median | -9.89 | -2.61 | -0.09 | -7.32 | -7.37 | Median | -3.51 | -23.96 | 10.64 | -29.55 |
| Min, Max | -55.69,  11.36 | -37.69,  35.92 | -41.86,  45.03 | -50.71,  34.30 | -40.20,  6.89 | Min, Max | -40.68,  54.35 | -43.92,  17.73 | -60.01,  123.58 | -66.32,  15.82 |
| **Timepoint 2** |  |  |  |  |  | **Timepoint 2** |  |  |  |  |
| n | 8 | 6 | 6 | 6 | 6 | n | 6 | 6 | 8 | 8 |
| Mean | -9.26 | 9.19 | 3.36 | -21.07 | -14.52 | Mean | 22.02 | 11.44 | 7.40 | 2.14 |
| SD | 19.80 | 24.87 | 30.14 | 11.65 | 24.11 | SD | 26.68 | 25.87 | 49.89 | 30.44 |
| Median | -1.24 | 18.72 | -3.42 | -19.32 | -9.89 | Median | 24.18 | 5.27 | 3.00 | -9.40 |
| Min, Max | -38.10,  17.37 | -24.76,  30.76 | -34.20,  41.21 | -36.88,  -3.07 | -45.22,  16.50 | Min, Max | -14.92,  56.88 | -8.17,  62.24 | -65.89,  93.77 | -26.10,  63.96 |
| **Timepoint 3** |  |  |  |  |  |  |  |  |  |  |
| n | 8 | 6 | 5 | 5 | 6 |  |  |  |  |  |
| Mean | 0.21 | 8.30 | 20.21 | -0.81 | -11.02 |  |  |  |  |  |
| SD | 11.80 | 14.24 | 27.40 | 14.97 | 10.15 |  |  |  |  |  |
| Median | 1.79 | 8.60 | 33.59 | -1.21 | -9.61 |  |  |  |  |  |
| Min, Max | -15.22,  18.85 | -14.88,  24.90 | -9.77,  44.78 | -19.73,  18.19 | -27.22,  -0.58 |  |  |  |  |  |
|  | | | | | |  | | | | |
| **GFAP**  % Change from BL | | | | | | **GFAP**  % Change from BL | | | | |
| **Timepoint 1** |  |  |  |  |  | **Timepoint 1** |  |  |  |  |
| n | 7 | 6 | 6 | 6 | 6 | n | 6 | 7 | 8 | 8 |
| Mean | -23.76 | -19.92 | 3.67 | -9.87 | -21.04 | Mean | 14.07 | -12.57 | 34.21 | -18.69 |
| SD | 30.22 | 62.54 | 16.95 | 34.09 | 33.86 | SD | 25.91 | 26.83 | 56.82 | 33.67 |
| Median | -19.35 | -0.00 | 4.50 | -9.89 | -19.41 | Median | 5.88 | -24.07 | 20.23 | -14.97 |
| Min, Max | -65.51,  8.93 | -137.78,  33.66 | -21.31,  23.34 | -52.15,  47.97 | -57.25,  23.57 | Min, Max | -7.28,  60.82 | -48.13,  32.10 | -23.05,  137.66 | -62.93,  37.64 |
| **Timepoint 2** |  |  |  |  |  | **Timepoint 2** |  |  |  |  |
| n | 8 | 6 | 6 | 6 | 6 | n | 6 | 6 | 8 | 8 |
| Mean | -15.11 | -8.43 | -10.55 | -1.01 | -4.89 | Mean | 36.01 | 0.79 | 34.46 | 6.77 |
| SD | 14.20 | 32.36 | 19.38 | 34.78 | 32.70 | SD | 23.52 | 17.88 | 57.19 | 24.37 |
| Median | -16.04 | -9.87 | -12.20 | -10.73 | -13.13 | Median | 32.15 | 0.47 | 10.06 | 2.72 |
| Min, Max | -34.91,  6.07 | -61.87,  26.18 | -36.49,  14.00 | -34.28,  60.84 | -34.15,  51.08 | Min, Max | 6.34,  67.56 | -19.41,  28.46 | -12.77,  145.53 | -30.48,  40.59 |
| **Timepoint 3** |  |  |  |  |  |  |  |  |  |  |
| n | 8 | 6 | 5 | 5 | 6 |  |  |  |  |  |
| Mean | 0.67 | -0.03 | 12.56 | 17.15 | -0.10 |  |  |  |  |  |
| SD | 17.52 | 34.52 | 15.64 | 21.74 | 26.61 |  |  |  |  |  |
| Median | -2.57 | -2.12 | 11.73 | 11.25 | -9.33 |  |  |  |  |  |
| Min, Max | -22.34,  26.99 | -50.92,  55.84 | -4.18,  32.66 | -3.78,  47.88 | -25.83,  42.04 |  |  |  |  |  |
|  | | | | | |  | | | | |
| **NfL**  % Change from BL | | | | | | **NfL**  % Change from BL | | | | |
| **Timepoint 1** |  |  |  |  |  | **Timepoint 1** |  |  |  |  |
| n | 7 | 6 | 6 | 6 | 6 | n | 6 | 7 | 8 | 8 |
| Mean | -12.47 | 7.00 | 2.39 | -1.61 | -15.19 | Mean | 0.37 | -6.52 | 12.52 | -14.86 |
| SD | 15.90 | 17.25 | 11.65 | 14.79 | 19.43 | SD | 14.59 | 14.36 | 61.05 | 27.91 |
| Median | -7.77 | 9.64 | 0.15 | 0.16 | -11.27 | Median | -3.23 | -7.12 | 5.93 | -16.49 |
| Min, Max | -39.69,  8.40 | -15.81,  28.95 | -8.95,  17.82 | -19.64,  21.39 | -43.91,  4.10 | Min, Max | -20.34,  21.74 | -26.94,  16.56 | -55.39,  106.46 | -58.98,  39.81 |
| **Timepoint 2** |  |  |  |  |  | **Timepoint 2** |  |  |  |  |
| n | 8 | 6 | 6 | 6 | 6 | n | 6 | 6 | 8 | 8 |
| Mean | 2.08 | 9.11 | 2.11 | -2.03 | 3.03 | Mean | 13.60 | 6.47 | 12.10 | 11.18 |
| SD | 12.19 | 15.93 | 25.44 | 15.67 | 20.53 | SD | 15.83 | 27.96 | 57.54 | 21.73 |
| Median | 3.78 | 4.55 | 4.23 | -5.75 | 8.37 | Median | 9.45 | 7.13 | 7.79 | 15.62 |
| Min, Max | -16.17,  23.51 | -4.42,  40.15 | -27.98,  36.91 | -19.07,  17.01 | -33.41,  26.77 | Min, Max | -6.92,  36.17 | -39.31,  46.15 | -56.55,  106.95 | -20.25,  35.69 |
| **Timepoint 3** |  |  |  |  |  |  |  |  |  |  |
| n | 8 | 6 | 5 | 5 | 6 |  |  |  |  |  |
| Mean | -2.93 | 27.52 | 2.84 | 69.18 | -2.03 |  |  |  |  |  |
| SD | 17.91 | 60.00 | 30.06 | 103.87 | 20.35 |  |  |  |  |  |
| Median | -8.72 | 13.24 | -6.25 | 4.37 | -6.13 |  |  |  |  |  |
| Min, Max | -21.34,  30.56 | -24.57,  138.52 | -25.54,  37.06 | -4.54,  233.51 | -27.19,  34.79 |  |  |  |  |  |

For SAD cohorts, timepoints 1, 2, and 3 were days 4, 21, and 140, respectively.

For MAD 10mg/kg Q4W cohort, timepoints 1 and 2 were days 70 and 196 (14 and 140 days after last dose), respectively.

For MAD 25mg/kg Q2W cohort, timepoints 1 and 2 were days 35 and 98 (7 and 70 days after last dose), respectively.

For MAD 60mg/kg Q4W cohort, timepoints 1 and 2 were days 63 and 126 (7 and 70 days after last dose), respectively.

Placebo samples for each MAD cohort were collected at the same time as the sabirnetug samples, and results were pooled.

GFAP, glial fibrillary acidic protein; Max. maximum; Min, minimum; NfL, neurofilament light; pTau181 or 217, tau phosphorylated at site 181 or 217; SD, standard deviation.

**Supplemental Table 12. Unpaired Student's T Test (two-tailed) Statistics for Percent Change in Plasma Biomarkers Results**

| **Plasma** **Biomarker** | **Timepoint** | **Comparison** **Group** **1** | **Comparison** **Group** **2** | **Mean** **Difference** | **t** **Statistic** | **Degrees** **of** **Freedom** | **p-value** |
| --- | --- | --- | --- | --- | --- | --- | --- |
| pTau181 | Timepoint 1 | ACU193 10 mg/kg Q4W | Placebo | -1.8053 | -0.13 | 11 | 0.8969 |
| pTau181 | Timepoint 1 | ACU193 25 mg/kg Q2W | Placebo | 5.1668 | 0.27 | 12 | 0.7906 |
| pTau181 | Timepoint 1 | ACU193 60 mg/kg Q4W | Placebo | -17.6754 | -1.64 | 12 | 0.1263 |
| pTau181 | Timepoint 2 | ACU193 10 mg/kg Q4W | Placebo | -4.4744 | -0.35 | 10 | 0.7329 |
| pTau181 | Timepoint 2 | ACU193 25 mg/kg Q2W | Placebo | -3.2772 | -0.15 | 12 | 0.8824 |
| pTau181 | Timepoint 2 | ACU193 60 mg/kg Q4W | Placebo | -6.8346 | -0.42 | 12 | 0.6808 |
|  | | | | | | | |
| pTau217 | Timepoint 1 | ACU193 10 mg/kg Q4W | Placebo | -18.2385 | -1.15 | 11 | 0.2760 |
| pTau217 | Timepoint 1 | ACU193 25 mg/kg Q2W | Placebo | 16.7456 | 0.64 | 12 | 0.5318 |
| pTau217 | Timepoint 1 | ACU193 60 mg/kg Q4W | Placebo | -26.4656 | -1.63 | 12 | 0.1298 |
| pTau217 | Timepoint 2 | ACU193 10 mg/kg Q4W | Placebo | -10.5806 | -0.70 | 10 | 0.5014 |
| pTau217 | Timepoint 2 | ACU193 25 mg/kg Q2W | Placebo | -14.6202 | -0.65 | 12 | 0.5295 |
| pTau217 | Timepoint 2 | ACU193 60 mg/kg Q4W | Placebo | -19.8784 | -1.27 | 12 | 0.2274 |
|  | | | | | | | |
| GFAP | Timepoint 1 | ACU193 10 mg/kg Q4W | Placebo | -26.6422 | -1.81 | 11 | 0.0972 |
| GFAP | Timepoint 1 | ACU193 25 mg/kg Q2W | Placebo | 20.1338 | 0.80 | 12 | 0.4384 |
| GFAP | Timepoint 1 | ACU193 60 mg/kg Q4W | Placebo | -32.7573 | -1.98 | 12 | 0.0714 |
| GFAP | Timepoint 2 | ACU193 10 mg/kg Q4W | Placebo | -35.2217 | -2.92 | 10 | 0.0153 |
| GFAP | Timepoint 2 | ACU193 25 mg/kg Q2W | Placebo | -1.5453 | -0.06 | 12 | 0.9517 |
| GFAP | Timepoint 2 | ACU193 60 mg/kg Q4W | Placebo | -29.2403 | -2.25 | 12 | 0.0437 |
|  | | | | | | | |
| NfL | Timepoint 1 | ACU193 10 mg/kg Q4W | Placebo | -6.8865 | -0.86 | 11 | 0.4104 |
| NfL | Timepoint 1 | ACU193 25 mg/kg Q2W | Placebo | 12.1581 | 0.47 | 12 | 0.6445 |
| NfL | Timepoint 1 | ACU193 60 mg/kg Q4W | Placebo | -15.2295 | -1.21 | 12 | 0.2496 |
| NfL | Timepoint 2 | ACU193 10 mg/kg Q4W | Placebo | -7.1301 | -0.54 | 10 | 0.5986 |
| NfL | Timepoint 2 | ACU193 25 mg/kg Q2W | Placebo | -1.4984 | -0.06 | 12 | 0.9520 |
| NfL | Timepoint 2 | ACU193 60 mg/kg Q4W | Placebo | -2.4136 | -0.23 | 12 | 0.8225 |

GFAP, glial fibrillary acidic protein; NfL, neurofilament light chain; pTau181 or 217, tau phosphorylated at site 181 or 217; tTau, total tau; Q2W, every 2 weeks; Q4W, every 4 weeks; VAMP2, vesicle-associated membrane protein 2

**Supplemental Figure 1. Correlation Plots in Which Cerebrospinal Fluid Samples were Shown to be Outliers**


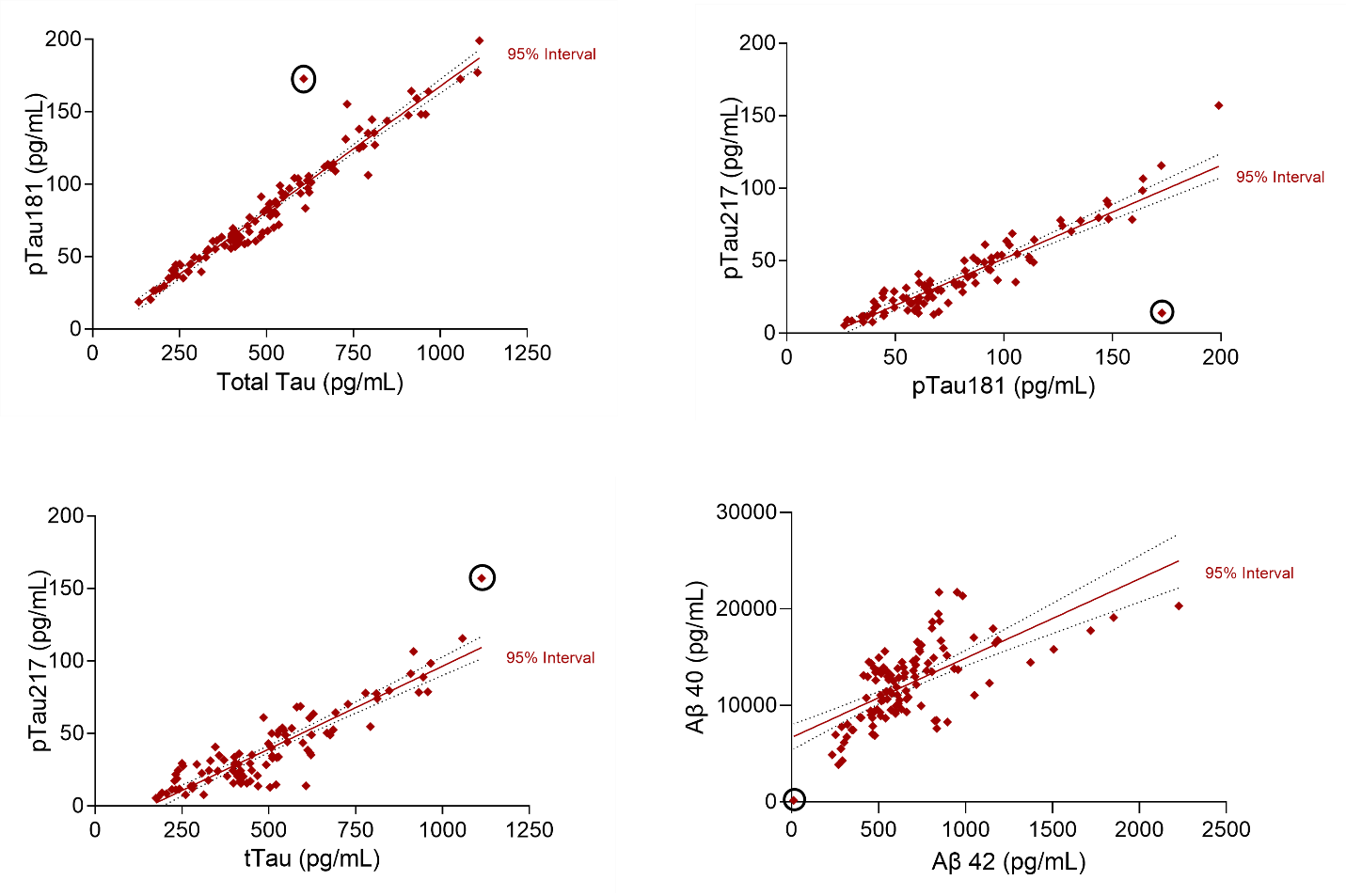


Aβ, amyloid-β; pTau181 or 217, tau phosphorylated at site 181 or 217; tTau, total tau.

**Supplemental Figure 2. Changes in Biomarker Concentrations in Cerebrospinal Fluid After a Single Dose of Sabirnetug**

The graphs show percent changes in CSF biomarker concentrations from baseline to Day 21 for participants in SAD cohorts 1-4 who received placebo or sabirnetug.

Aβ, amyloid-β; CSF, cerebrospinal fluid; NPTX2, neuronal pentraxin 2; PBO, placebo; pTau, phosphorylated tau protein; Q2W, every 2 weeks; Q4W, every 4 weeks, SAD, single ascending dose; tTau, total tau protein; VAMP2, vesicle-associated membrane protein 2

**Supplemental Figure 3. Correlations between Plaque Reduction and Phosphorylated Tau in Cerebrospinal Fluid**


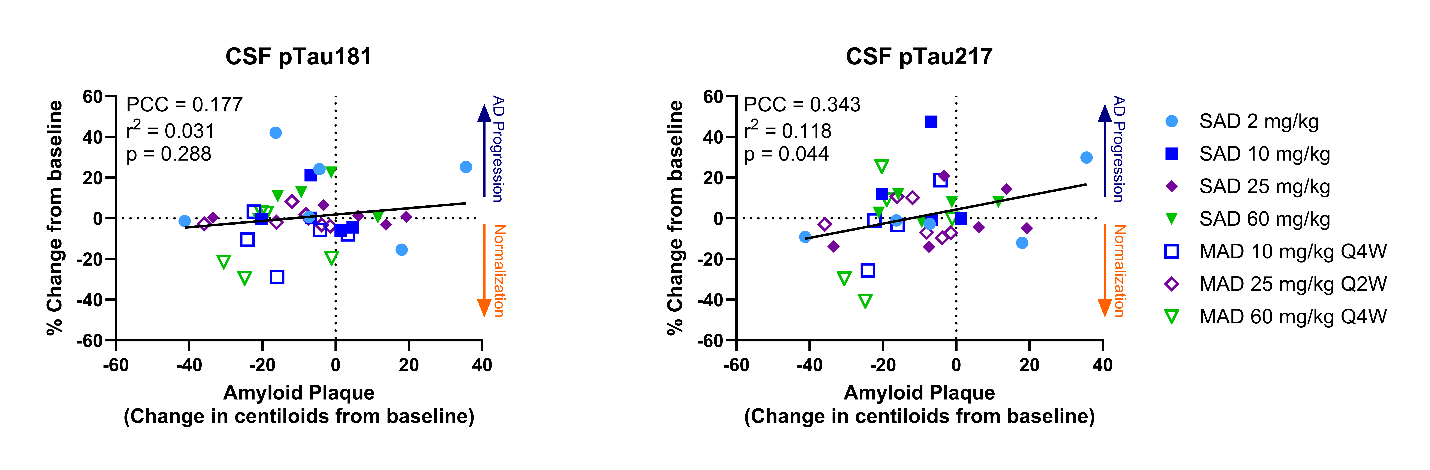


Correlation plots present data points from individual subjects; lines represent linear regressions

CSF, cerebrospinal fluid; MAD, multiple ascending dose; pTau181 or 217, tau phosphorylated at site 181 or 217; Q2W, every 2 weeks; Q4W, every 4 weeks; SAD, single ascending dose

References

1. Gobom J, Parnetti L, Rosa-Neto P, et al. Validation of the LUMIPULSE automated immunoassay for the measurement of core AD biomarkers in cerebrospinal fluid. Clin Chem Lab Med. 2022;60(2):207-19.

2. Willemse EAJ, Tijms BM, van Berckel BNM, et al. Comparing CSF amyloid-beta biomarker ratios for two automated immunoassays, Elecsys and Lumipulse, with amyloid PET status. Alzheimers Dement (Amst). 2021;13(1):e12182.

3. De Vos A, Bjerke M, Brouns R, et al. Neurogranin and tau in cerebrospinal fluid and plasma of patients with acute ischemic stroke. BMC Neurol. 2017;17(1):170.

4. Aveneau C, Hourregue C, Cognat E, et al. Cerebrospinal fluid neurogranin in Alzheimer's disease studies: are immunoassay results interchangeable? Clin Chem Lab Med. 2022;60(1):e13-e7.

5. Lambrechts C, Van Loo M, Vanbrabant J, et al. Performance of optimized prototype LUMIPULSE G immunoassays for plasma pTau181 and pTau217. AAIC. Alzheimer's Dement. 2023;19:e082944.

6. Das S, Goossens J, Jacobs D, et al. Synaptic biomarkers in the cerebrospinal fluid associate differentially with classical neuronal biomarkers in patients with Alzheimer's disease and frontotemporal dementia. Alzheimers Res Ther. 2023;15(1):62.

7. Galasko D, Xiao M, Xu D, et al. Synaptic biomarkers in CSF aid in diagnosis, correlate with cognition and predict progression in MCI and Alzheimer's disease. Alzheimers Dement. 2019;5:871-82.

8. Chatterjee P, Pedrini S, Doecke JD, et al. Plasma Aβ42/40 ratio, p-tau181, GFAP, and NfL across the Alzheimer's disease continuum: A cross-sectional and longitudinal study in the AIBL cohort. Alzheimers Dement. 2023;19(4):1117-34.

9. Wojdała AL, Bellomo G, Toja A, et al. CSF and plasma Aβ42/40 across Alzheimer’s disease continuum: comparison of two ultrasensitive Simoa^®^ assays targeting distinct amyloid regions. Clin Chem Lab Med. 2024;62(2):332-40.

10. Bayoumy S, Verberk IMW, den Dulk B, et al. Clinical and analytical comparison of six Simoa assays for plasma P-tau isoforms P-tau181, P-tau217, and P-tau231. Alzheimers Res Ther. 2021;13(1):198.

11. Ashton NJ, Brum WS, Di Molfetta G, et al. Diagnostic Accuracy of a Plasma Phosphorylated Tau 217 Immunoassay for Alzheimer Disease Pathology. JAMA Neurol. 2024;81(3):255-63.
